# Testing the applicability of a governance checklist for high-risk AI-based learning outcome assessment in Italian universities under the EU AI act annex III

**DOI:** 10.3389/frai.2025.1718613

**Published:** 2025-12-11

**Authors:** Flavio Manganello, Alberto Nico, Martina Ragusa, Giannangelo Boccuzzi

**Affiliations:** 1 Institute for Educational Technology, National Research Council, Genoa, Italy; 2 Department of Law, University “Aldo Moro”, Bari, Italy; 3 Department of Education Studies, University of Bologna “Alma Mater Studiorum”, Bologna, Italy

**Keywords:** artificial intelligence in education, AI-based learning outcome assessment, explainable AI, ALTAI, educational evaluation, transparency, accountability, AI governance

## Abstract

**Background:**

The EU AI Act classifies AI-based learning outcome assessment as high-risk (Annex III, point 3b), yet sector-specific frameworks for institutional self-assessment remain underdeveloped. This creates accountability gaps affecting student rights and educational equity, as institutions lack systematic tools to demonstrate that algorithmic assessment systems produce valid and fair outcomes.

**Methods:**

This exploratory study tests whether ALTAI’s trustworthy AI requirements can be operationalized for educational assessment governance through the XAI-ED Consequential Assessment Framework, which integrates three educational evaluation theories (Messick’s consequential validity, Kirkpatrick’s four-level model, Stufflebeam’s CIPP). Following pilot testing with three institutions, four independent coders applied a 27-item checklist to policy documents from 14 Italian universities (13% with formal AI policies plus one baseline case) using four-point ordinal scoring and structured consensus procedures.

**Results:**

Intercoder reliability analysis revealed substantial agreement (Fleiss’s *κ* = 0.626, Krippendorff’s *α* = 0.838), with higher alpha reflecting predominantly adjacent-level disagreements suitable for exploratory validation. Analysis of 14 universities reveals substantial governance heterogeneity among early adopters (Institutional Index: 0.00–60.32), with Technical Robustness and Safety showing lowest implementation (M = 19.64, SD = 21.08) and Societal Well-being highest coverage (M = 52.38, SD = 29.38). Documentation prioritizes aspirational statements over operational mechanisms, with only 13% of Italian institutions having adopted AI policies by September 2025.

**Discussion:**

The framework demonstrates feasibility for self-assessment but reveals critical misalignment: universities document aspirational commitments more readily than technical safeguards, with particularly weak capacity for validity testing and fairness monitoring. Findings suggest three interventions: (1) ministerial operational guidance translating EU AI Act requirements into educational contexts, (2) inter-institutional capacity-building addressing technical-pedagogical gaps, and (3) integration of AI governance indicators into national quality assurance systems to enable systematic accountability. The study contributes to understanding how educational evaluation theory can inform the translation of abstract trustworthy AI principles into outcome-focused institutional practices under high-risk classifications.

## 1 Introduction

The European Union AI Act (Regulation 2024/1689) classifies AI systems used for evaluating learning outcomes in educational and vocational institutions as high-risk applications (Annex III, point 3b). This classification recognizes that algorithmic mediation in student assessment affects fundamental rights, equity, and access to future opportunities. However, empirical evidence reveals a disconnect between regulatory expectations and institutional readiness. A survey of over 900 higher education technology professionals found that only 23% of institutions have AI-related acceptable use policies, while 48% report lacking appropriate guidelines for ethical and effective decision-making about AI use (Robert and McCormack, 2024).

This governance challenge reflects broader patterns across higher education systems worldwide. A 2023 survey across six European countries found that 80% of participating institutions lacked AI policies or were uncertain whether policies existed (Media and Learning Association, 2023). Recent evidence suggests gradual improvement: a 2025 UNESCO global survey found that 19% of institutions had formal AI policies, with 42% developing guidance frameworks, though regional variation persists (UNESCO, 2025).

Comparative analysis of 15 policies across eight European countries reveals substantial heterogeneity, with gaps particularly evident in data privacy protections and equitable access provisions (Stracke et al., 2025). Governance adoption appears stratified by institutional resources: 82% of the world’s top 50 universities had GenAI guidelines by late 2024 (Kontogiannis et al., 2024), substantially higher than comprehensive national systems.

This resource stratification suggests that comprehensive national higher education systems face governance challenges reflecting systemic capacity constraints rather than institutional or national anomalies. Yet regardless of resources, all institutions confront a common gap: translating abstract regulatory requirements into operational accountability mechanisms.

Against this backdrop of fragmented global adoption, regulatory timelines create urgency. The AI Act entered into force on 1 August 2024, with full applicability by 2 August 2026, yet mechanisms for demonstrating compliance with high-risk classification requirements remain underdeveloped (European Parliament, Council of the European Union, 2024).

Existing tools address partial dimensions without providing integrated solutions. The Assessment List for Trustworthy AI (ALTAI) offers structured self-assessment (European Commission, Directorate-General for Communications Networks, Content and Technology, 2020) but lacks integration with educational evaluation practices (Fedele et al., 2024). Similarly, explainability research demonstrates technical sophistication but often does not produce explanations meaningful for learners and educators (Albaladejo-González et al., 2024; Hooshyar and Yang, 2024).

These gaps converge on a central challenge: translating abstract regulatory requirements into pedagogically grounded governance practices that institutions can operationalize.

Educational evaluation theory provides conceptual resources for addressing these gaps. Three paradigms directly address outcome-focused accountability needs: Messick’s (1995) consequential validity emphasizes social and ethical consequences of assessment practices, Kirkpatrick and Kirkpatrick’s (2006) four-level model enables multi-temporal impact evaluation, and Stufflebeam’s (1983) CIPP framework structures contextual institutional assessment.

Integrating these evaluation paradigms with ALTAI’s trustworthy AI requirements shifts governance from compliance verification toward pedagogical accountability, enabling institutions to demonstrate not only that AI systems meet technical standards but that they produce valid and equitable learning outcomes.

This study pursues two interrelated objectives. First, it conducts exploratory validation of the XAI-ED Consequential Assessment Framework (CAF), developed in our previous theoretical work (Manganello et al., 2025), testing whether ALTAI requirements can be operationalized for institutional self-assessment through educational evaluation theory.

Second, it provides baseline empirical evidence on AI governance documentation in Italian higher education, a comprehensive national system that exemplifies the resource-stratified adoption patterns documented globally. The study examines all 13 universities that had formally adopted AI policies by September 2025 (13% of institutions, consistent with 19% global adoption rates), plus one baseline case without policy documentation.

This complete enumeration strategy is methodologically appropriate for exploratory validation. Early-adopting institutions test governance frameworks under real-world resource constraints, enabling instrument validation while revealing implementation challenges. Although early adopters likely possess greater technical capacity and regulatory awareness than the broader population (limiting generalizability to sector-wide patterns) their governance approaches establish baseline models that later adopters will likely adapt as the 2026 regulatory deadline approaches.

The study is guided by two research questions:

- RQ1 (feasibility and reliability): To what extent can the XAI-ED CAF checklist be applied consistently across Italian universities’ documents?
- RQ2 (policy coverage and patterns): Which XAI-ED CAF dimensions are most and least documented in the publicly available policies of early-adopting universities, as measured by normalized XAI-ED CAF scores?

This study advances AI governance scholarship in three ways. Methodologically, it demonstrates that ALTAI can be operationalized for institutional self-assessment when integrated with educational evaluation theory, providing a validated checklist (Krippendorff’s *α* = 0.838) suitable for exploratory governance assessment. Empirically, it establishes baseline evidence that only 13% of Italian universities have adopted AI policies by September 2025, with substantial heterogeneity in governance approaches (Institutional Index: 0.00–60.32) and systematic gaps in technical robustness documentation. Theoretically, it reveals universities as regulatory laboratories where abstract compliance requirements are tested against organizational realities, documenting misalignment between regulatory expectations of technical validation capacity and institutions’ actual governance capabilities.

These findings carry implications for educational equity and regulatory design. Without systematic outcome evaluation, AI-mediated assessment decisions affecting student progression risk escaping meaningful oversight, with vulnerable populations facing particular harm when algorithmic evaluations lack validity and fairness safeguards. The framework provides institutions with structured self-assessment tools, yet empirical evidence reveals substantial gaps between what the EU AI Act expects universities to demonstrate and what their current governance capacities enable. Bridging this gap requires not only better frameworks but sector-specific implementation guidance, capacity-building mechanisms, and integration of AI governance into existing quality assurance systems, interventions this study specifies based on documented institutional needs.

## 2 Theoretical framework

The present study builds upon recent theoretical advances in the governance of AIB-LOA. In this study, AIB-LOA is understood as the set of practices where artificial intelligence systems contribute directly to the evaluation of student performance. The EU AI Act (Regulation 2024/1689, Annex III, 3b) explicitly classifies as high-risk those systems used for the assessment of learning outcomes, such as automated scoring of written exams, algorithmic grading of coursework, or adaptive testing procedures that determine certification. Beyond these regulatory definitions, AIB-LOA also encompasses institutional practices where AI is employed to generate exam questions, support the construction of grading rubrics, or provide automated feedback that influences marks and progression.

Manganello et al. (2025) developed the XAI-ED CAF as a sector-specific, outcome-focused governance model that reinterprets the ALTAI through established educational evaluation theories. The present study operationalizes this theoretical framework into an empirically testable checklist for document analysis, examining its applicability in institutional contexts.

AI systems in educational assessment function as regulatory instruments (Reidenberg, 1997), directly mediating decisions affecting student rights and opportunities. The EU AI Act classifies educational assessment systems as high-risk (Annex III, point 3b) but does not specify mechanisms for evaluating whether AI-mediated assessments produce valid, fair, and educationally meaningful results over time. This study addresses this gap by examining how universities translate regulatory obligations into outcome-focused governance practices for AIB-LOA.

### 2.1 ALTAI and the problem of educational translation

ALTAI provides seven requirements for trustworthy AI: human agency and oversight, technical robustness and safety, privacy and data governance, transparency, diversity and fairness, societal well-being, and accountability (European Commission, Directorate-General for Communications Networks, Content and Technology, 2020). While offering value as a self-assessment tool, ALTAI remains compliance-oriented and lacks integration with educational validity and outcome assessment.

This limitation has been documented through multiple analytical perspectives. Radclyffe et al. (2023) observe that ALTAI does not differentiate risks, lacks peer-comparison mechanisms, and omits integration with fundamental rights impact assessments. Peterson and Broersen (2024) argue that automated systems cannot autonomously provide normative justifications, only propositional explanations of outputs, rendering “explainable ethical AI” problematic as a governance goal. Fedele et al. (2024) applied ALTAI to student performance prediction systems, identifying vulnerabilities but not exploring adaptation to AIB-LOA specifically designated as high-risk.

The XAI-ED CAF addresses these limitations by embedding ALTAI within three complementary theories of educational evaluation. Messick’s (1995) consequential validity emphasizes that validity includes not only technical accuracy but also social and ethical consequences of assessment practices. Kirkpatrick and Kirkpatrick’s (2006) four-level model structures evaluation across reaction, learning, behavior, and results. Stufflebeam’s (1983) CIPP framework provides systematic evaluation across context, inputs, processes, and products. Through this integration, the framework shifts from technical system assessment toward pedagogical outcome accountability.

### 2.2 Framework operationalization and scope

The XAI-ED CAF operationalizes ALTAI as a document analysis checklist enabling systematic evaluation of how universities publicly document AIB-LOA governance across policy texts, regulations, and official statements. This operationalization required strategic adaptations. While ALTAI encompasses approximately 80–90 operational questions across detailed technical subcategories, the XAI-ED CAF prioritizes institutional policy articulation over implementation specifics.

The adaptation emphasizes pedagogical accountability over operational detail. Where ALTAI addresses technical vulnerabilities such as data poisoning and adversarial attacks, the XAI-ED CAF focuses on institutional commitments to construct validity, educational meaningfulness, and learning outcome alignment (Messick, 1995). The checklist consolidates related requirements into coherent governance constructs reflecting how universities typically organize policy frameworks, thereby supporting outcome-focused educational accountability rather than technical system audit.

This scope limitation acknowledges that publicly accessible policy documents cannot systematically capture operational details such as actual cybersecurity protocols, stakeholder consultation effectiveness, or real-world bias mitigation outcomes. However, policy documentation constitutes legally and institutionally significant evidence. Formal governance frameworks establish accountability structures, create procedural obligations, and provide foundations for oversight and redress mechanisms. Document analysis therefore captures an essential dimension of governance (the institutional capacity to articulate, formalize, and publicly commit to governance standards), which represents a necessary though insufficient condition for effective AI accountability in educational assessment.

### 2.3 Human-AI interaction: theory of mind and metacognitive capabilities

Effective governance of AIB-LOA requires consideration of how AI systems interact with human stakeholders in educational contexts. Recent advances in AI research emphasize that systems deployed in socially complex environments, such as educational assessment, require capabilities extending beyond technical accuracy to include social intelligence and self-regulation mechanisms (Bamicha and Drigas, 2024a; Williams et al., 2022).

Theory of Mind (ToM), defined as the ability to attribute mental states, beliefs, and intentions to others, constitutes a foundational component of human social intelligence (Williams et al., 2022). In educational AI systems, artificial ToM capabilities would enable systems to model learner knowledge states, interpret instructor intentions, and adapt explanations based on stakeholder understanding levels. Williams et al. (2022) argue that artificial social intelligence grounded in ToM principles supports transparent communication with humans, improving trust in AI systems by enabling users to better predict system behavior based on their understanding of system capabilities and constraints. This transparency dimension directly connects to ALTAI’s requirements for human agency and oversight, as systems capable of modeling user mental states can provide contextually appropriate explanations calibrated to user expertise.

Complementing ToM, metacognition (i.e., the capacity for self-monitoring, evaluation, and regulation of cognitive processes) enables AI systems to assess their own performance limitations and uncertainty (Bamicha and Drigas, 2024a). In educational assessment contexts, metacognitive capabilities would allow AI systems to recognize when confidence in scoring decisions falls below acceptable thresholds, identify when assessment items may be ambiguous or culturally biased, and determine when human oversight is necessary.

Bamicha and Drigas (2024b) demonstrate that social robots equipped with ToM and metacognitive functions can engage in safer and more satisfactory interactions with humans when their design incorporates ethical constraints and self-evaluation mechanisms. Applied to AIB-LOA, these capabilities would support the technical robustness and safety requirements by enabling systems to introspectively monitor their decision-making processes and communicate uncertainty to human stakeholders.

The integration of ToM and metacognitive capabilities into AI governance frameworks addresses a critical gap between technical system functionality and pedagogical accountability. Current AIB-LOA systems often lack mechanisms for interpreting educator intentions, modeling learner understanding, or self-assessing decision reliability. The XAI-ED CAF’s emphasis on transparency and human oversight creates space for such capabilities, but institutional governance documentation examined in this study reveals limited attention to these human-AI interaction dimensions. Universities document aspirational commitments to explainability but rarely specify how AI systems should model stakeholder knowledge or communicate uncertainty. This suggests that governance frameworks must evolve beyond compliance-oriented transparency toward interaction-focused accountability that recognizes AI systems as social agents operating within complex educational ecologies. Future research should examine how ToM and metacognitive capabilities can be operationalized in educational AI governance standards and assessed through institutional policy frameworks.

## 3 Methods

### 3.1 Study design and sampling

This study adopts a cross-sectional document analysis design with the primary aim of conducting exploratory validation of the XAI-ED CAF checklist. The objective is to test whether checklist categories and items are applicable in institutional practice rather than to provide a comprehensive mapping of AI governance across Italian higher education.

The study population comprised 100 legally recognized Italian universities as defined by the official Ministry of Universities and Research registries: 61 state universities, 8 special-regulation university institutes, 20 non-state institutions, and 11 distance-learning universities (Ministero dell’Università e della Ricerca, 2025). A systematic web-based screening was conducted between June and September 2025 to determine whether each institution had at least one publicly available policy or guideline explicitly addressing artificial intelligence. Screening targeted website sections most likely to contain governance materials (statutes, regulations, transparency documentation, quality assurance reports, digital transformation strategies, and official communications) using Google’s Advanced Search with bilingual structured queries. The outcome of this phase was binary at the institutional level (policy present/absent). The screening identified 13 universities (13%) with formal AI-related policies accessible online. The complete screening dataset covering all 100 universities is provided in Supplementary Material 1.

Given the exploratory scope and the low population-level adoption rate (13%), the study adopted a complete enumeration strategy including all 13 institutions with an active policy. This approach differs from studies focusing on elite institutions (e.g., Kontogiannis et al., 2024, analyzing top 50 global universities) by capturing the full range of early-adopting institutions within a comprehensive national system, providing insights into governance patterns beyond resource-advantaged contexts. Additionally, one university without any AI-related policy (Università degli Studi di Napoli “Federico II,” UNINA) was purposively included as a baseline case, to test checklist sensitivity under absence-of-evidence conditions. The resulting analytical sample thus comprised 14 universities. Institutional characteristics were not used for stratification but are reported in Table 1 to provide contextual information on the sample composition (size, geographical distribution, and legal status).

**Table 1:** Italian universities with active AI policies (as of August 2025).

University	Region	Size	Legal Status	Policy URL	Last update
Università degli Studi di Teramo (UNITE)	Abruzzo	Small (<10 k)	Public	URL	2025–03
Alma Mater Studiorum - Università di Bologna (UNIBO)	Emilia-Romagna	Large (>40 k)	Public	URL	2025–01
Università degli Studi di Parma (UNIPR)	Emilia-Romagna	Medium (10–40 k)	Public	URL	2025–05
Università degli Studi di Bergamo (UNIBG)	Lombardia	Medium (10–40 k)	Public	URL	2025–06
Università degli Studi di Milano (UNIMI)	Lombardia	Large (>40 k)	Public	URL	2024–12
Politecnico di Milano (POLIMI)	Lombardia	Large (>40 k)	Public	URL	2025–03
Università degli Studi di Camerino (UNICAM)	Marche	Small (<10 k)	Public	URL	2024–07
Università degli Studi del Piemonte Orientale “Amedeo Avogadro” (UNIPO)	Piemonte	Medium (10–40 k)	Public	URL	2025–02
Università degli Studi di Firenze (UNIFI)	Toscana	Large (>40 k)	Public	URL	2025–03
Università degli Studi di Siena (UNISI)	Toscana	Medium (10–40 k)	Public	URL	2023–09
Università degli Studi di Trento (UNITN)	Trentino-Alto Adige	Medium (10–40 k)	Public	URL	2025–03
Università per Stranieri di Perugia (UNISTRAPG)	Umbria	Small (<10 k)	Public	URL	2025–04
Università Ca′ Foscari Venezia (UNIVE)	Veneto	Medium (10–40 k)	Public	URL	2025–09

### 3.2 Checklist development

The translation of XAI-ED CAF theoretical framework into an operational checklist required systematic operationalization of illustrative indicators presented in the conceptual study (Manganello et al., 2025). Each of the seven XAI-ED CAF dimensions was mapped against its corresponding pedagogical foundation. Illustrative indicators were systematically decomposed into constituent policy elements identifiable through document analysis.

Each checklist item was formulated as an evaluative question scored on a four-point ordinal scale: 0 (no evidence found), 1 (weak evidence with vague references), 2 (partial implementation with explicit policy statements but limited operationalization), and 3 (documented implementation with verifiable evidence). The decomposition process yielded a checklist structure in which six dimensions are operationalized through four items each, while one dimension comprises three items, resulting in 27 items total across the seven ALTAI dimensions. The full checklist, including items and scoring instructions, is provided in Supplementary Material 2.

### 3.3 Pilot study and reliability establishment

The coding team consisted of four researchers with complementary expertise: educational technology and AI in education (FM, 15 years of experience), explainable AI and algorithmic transparency (MR, 4 years), education policy and institutional governance (GB, 5 years), and data protection law and digital rights (AN, 7 years). All coders held or were pursuing doctoral qualifications and had prior experience in systematic document analysis.

Before data collection, the team participated in a two-session training phase (totaling six hours) covering the XAI-ED CAF theoretical framework, the checklist structure, scoring guidelines, and the consensus-building protocol. Training also included joint practice on sample documents to ensure common interpretation of item formulations and score thresholds.

A pilot study was then conducted on a subsample of three universities purposively selected to represent diversity in institutional size and typology. For these universities, a full document harvest was carried out to test both the comprehensiveness of the retrieval strategy and the clarity of checklist operationalization. All coders independently applied the checklist to the retrieved policy corpus, documenting for each item the evidence supporting their assigned score, including quotations, document titles, section references, and rationale for the chosen level.

After independent coding, the team met in a structured debriefing session to identify and discuss systematic discrepancies in evidence interpretation. Disagreements were traced to ambiguous phrasing in a small number of items, which were consequently refined for greater operational precision. Scoring guidelines were updated to specify when general references to digital ethics or data protection could be considered partial evidence of AI-related governance.

Preliminary reliability analysis on the pilot dataset showed substantial agreement. Pairwise Cohen’s kappa values ranged from 0.668 to 0.946 (average *κ* = 0.806). The overall Fleiss’s kappa across all four coders was 0.806 (95% CI: 0.749–0.864), and Krippendorff’s alpha reached 0.913 (95% CI: 0.864–0.949), exceeding the conventional 0.80 threshold and approaching the 0.90 benchmark suggested for high-stakes decision-making contexts (Krippendorff, 2018; Landis and Koch, 1977; McHugh, 2012). These results indicated internal consistency and interpretive stability of the coding protocol before proceeding to the full-scale analysis.

### 3.4 Main study coding procedure

Following completion of the pilot, the document harvest was extended to the remaining 11 universities in the analytical sample, using the refined checklist and standardized retrieval protocol. The extended collection was completed within the same overall window (June–September 2025), with a data-freeze at the end of September 2025.

For universities with an identified AI policy, coders systematically collected all related documentation, logging retrieval dates, URLs, document types and titles, and preliminary relevance assessments. For the baseline institution (UNINA), the same search protocol was applied to verify the absence of policy documentation, confirming the completeness of the screening outcome.

Coders worked independently and in parallel. After independent retrieval, document inventories were cross-checked; any document found by one coder but missed by others was circulated for joint eligibility verification before inclusion. Using the harvested corpus, coders then applied the checklist independently. For each non-zero score, coders cited exact textual evidence, including the document title, section, and justification.

Following independent scoring, the team compared the four score matrices and conducted structured review sessions to resolve discrepancies through collective discussion of the underlying evidence.

The consensus resolution process resulted in score changes for approximately 43.4% of item-institution combinations (164 of 378 total assessments). The majority of changes (99.4%) involved adjacent score levels, indicating threshold differences rather than divergent interpretive frameworks.

#### 3.4.1 Data analysis

To evaluate feasibility and reliability (RQ1), intercoder reliability was assessed using two complementary metrics: Fleiss’s Kappa and Krippendorff’s Alpha. These metrics measure agreement among multiple raters but differ in their treatment of ordinal scales. Fleiss’s kappa treats all disagreements equally regardless of magnitude, while Krippendorff’s alpha penalizes disagreements proportionally to their distance on the ordinal scale (Cohen, 1960; Fleiss, 1971; Krippendorff, 2004). Both metrics were calculated using the ordinal method appropriate for the four-point scoring scale (0–3).

The minimum acceptable thresholds were set following established guidelines: *κ* > 0.60 for “substantial agreement” (Landis and Koch, 1977) and *α* > 0.80 for “acceptable agreement” in exploratory instrument validation contexts (Krippendorff, 2004). Reliability analysis was conducted at both item and dimension levels. Confidence intervals (95%) were calculated using bootstrap resampling methods to assess estimation precision.

To examine policy coverage patterns (RQ2), descriptive analysis was conducted using normalized dimension scores on a 0–100 scale. For each institution, raw item scores were aggregated at dimension level by calculating the mean of all items within each dimension (range 0–3), then normalized using the formula:


$$\text{Scor}e_{d}^{\left(0-100\right)}=(\frac{{\overset{-}{x}}_{d}}{3}\;)\times 100$$

where ${\overset{-}{x}}_{d}$ is the average of all items in dimension d . An Institutional Index was computed as the unweighted mean of all seven normalized dimension scores.

Mean scores and standard deviations were calculated for each XAI-ED CAF dimension across all institutions. Dimensions were ranked by mean score to identify which governance areas showed higher or lower levels of documentation. Quantitative findings were supplemented by qualitative interpretation of coding notes to contextualize numerical patterns within institutional governance approaches.

### 3.5 Ethical considerations

The study relies exclusively on publicly accessible institutional documents. No personal or sensitive data were collected. As such, the study falls outside the scope of human subject research and does not require ethics committee approval. Data collection complied with the principles of GDPR regarding the use of publicly available institutional information.

## 4 Results

### 4.1 Applicability and reliability (RQ1)

Intercoder reliability assessment yielded two complementary metrics: Fleiss’s *κ* = 0.626 (95% CI: 0.598–0.654) and Krippendorff’s *α* = 0.838 (95% CI: 0.805–0.865). Both metrics measure agreement among multiple raters, but they differ in how they handle ordinal data and disagreement severity.

Fleiss’s kappa treats all disagreements equally, whether coders disagree by one point (scoring 1 versus 2) or by three points (scoring 0 versus 3). Krippendorff’s alpha, by contrast, penalizes disagreements proportionally to their magnitude on the ordinal scale, giving less weight to adjacent-level disagreements and more weight to extreme disagreements. This methodological difference explains the apparent discrepancy between the two values.

Table 2 presents interpretation guidelines for both reliability metrics alongside the study values. According to conventional guidelines (Landis and Koch, 1977; Krippendorff, 2004), Fleiss’s *κ* = 0.626 indicates “substantial agreement” (threshold: 0.61–0.80), while Krippendorff’s α = 0.838 reaches “acceptable agreement” (threshold: 0.81–0.99). The higher Krippendorff’s alpha value reflects the fact that disagreements predominantly involved adjacent score levels (scoring 1 versus 2) rather than extreme disagreements (0 versus 3). Coders shared similar policy interpretations but differed on threshold judgments, determining whether evidence was “weak” (score 1) or “partial” (score 2) rather than fundamentally disagreeing about policy presence or absence. For exploratory instrument validation in governance contexts, both values are deemed acceptable, with the pattern of disagreement suggesting the need for refined scoring guidelines rather than conceptual incoherence.

**Table 2:** Interpretation guidelines for reliability coefficients.

Metric assessment	Range	Interpretation	Study Value	Assessment
Fleiss’s κ	< 0.00	Poor agreement	0.626 (95% CI: 0.598–0.654)	Substantial agreement (acceptable for exploratory validation)
	0.00–0.20	Slight agreement		
	0.21–0.40	Fair agreement		
	0.41–0.60	Moderate agreement		
	0.61–0.80	Substantial agreement		
	0.81–1.00	Almost perfect agreement		
Krippendorff’s α	< 0.66	Unacceptable agreement	0.838 (95% CI: 0.805–0.865)	Acceptable agreement (exceeds threshold for exploratory use)
	0.66–0.80	Tentatively acceptable		
	0.81–0.99	Acceptable agreement		
	1.00	Perfect agreement		

Guidelines from Landis and Koch (1977) for Fleiss’s κ and Krippendorff (2004) for α. Both metrics assess agreement among four independent coders across 27 checklist items applied to 14 institutions (378 total assessments).

Coders showed strong consensus for boundary categories: absent policies (score 0: *κ* = 0.774, 95% CI: 0.733–0.815) and documented implementations (score 3: κ = 0.694, 95% CI: 0.653–0.735). Agreement was moderate for intermediate levels (score 1: κ = 0.505; score 2: κ = 0.508). This pattern is methodologically significant. The strong agreement on boundary categories (scores 0 and 3) indicates that coders consistently identified both policy absence and robust documentation: the most consequential distinctions for governance assessment under the EU AI Act, where binary presence or absence of mandated safeguards determines regulatory compliance. The moderate agreement on intermediate scores reflects the challenge of distinguishing aspirational statements from partial implementation in policy texts, a substantive governance issue rather than a coding artifact.

Cohen’s kappa for pairwise comparisons ranged from 0.438 to 0.922 (mean = 0.624), with highest agreement between coders with technical-pedagogical backgrounds (FM-MR: κ = 0.922) and lowest between governance and technical perspectives (GB-MR: κ = 0.438). This variation suggests a challenge: disciplinary background influences policy interpretation. If legal scholars and computer scientists systematically interpret the same policy text differently, governance frameworks require either stronger operational definitions or explicit recognition that accountability is inherently multi-perspectival.

Item-level analysis identified differential reliability patterns. Items addressing privacy compliance (GDPR-aligned language) and human agency mechanisms (established quality assurance terminology) achieved higher consistency. Items concerning technical robustness, validity testing procedures, and fairness monitoring showed lower reliability, reflecting either ambiguous coding criteria or institutional variation in documenting these dimensions.

### 4.2 Policy coverage (RQ2)

The sampling procedure identified 13 institutions (13% of 100 Italian universities) with formally adopted AI policies by September 2025. This adoption rate is consistent with broader European patterns documented by the Media and Learning Association (2023), where 80% of surveyed institutions lacked AI policies, and aligns with the 19% global adoption rate reported by UNESCO (2025). However, it contrasts sharply with the 82% adoption rate observed among the world’s top 50 universities (Kontogiannis et al., 2024), suggesting that governance adoption is stratified by institutional resources and that comprehensive national systems lag substantially behind elite, research-intensive institutions. This low adoption rate indicates that formal AI governance remains exceptional rather than normative in Italian higher education. Within this context of limited sector-wide adoption, analysis of these 13 policy-adopting institutions plus one baseline case reveals substantial variation in documented governance approaches. Given the exploratory nature of this early-adopter sample, findings characterize governance experimentation among policy pioneers rather than established sector-wide practices.

Table 3 presents dimension-level scores. Societal and Environmental Well-being showed highest coverage (M = 52.38, SD = 29.38), followed by Human Agency and Oversight (M = 47.62, SD = 19.46) and Privacy and Data Governance (M = 34.52, SD = 17.56). Technical Robustness and Safety showed lowest documented implementation (M = 19.64, SD = 21.08), with Fairness and Educational Equity similarly underdeveloped (M = 22.62, SD = 18.90).

**Table 3:** Descriptive statistics for XAI-ED CAF governance dimensions (*N* = 14).

XAI-ED CAF dimension	Mean	SD	Min	Max	Median	Coverage level
Societal & environmental well-being	52.38	29.38	0.00	88.89	50.00	Medium-High
Human agency & oversight	47.62	19.46	0.00	75.00	50.00	Medium
Privacy & data governance	34.52	17.56	0.00	75.00	33.33	Medium
Transparency	30.95	17.12	0.00	58.33	33.33	Medium
Accountability	26.79	20.20	0.00	66.67	25.00	Low-Medium
Fairness & educational equity	22.62	18.90	0.00	50.00	20.84	Low
Technical robustness & safety	19.64	21.08	0.00	58.33	16.67	Low

**Figure 1:**
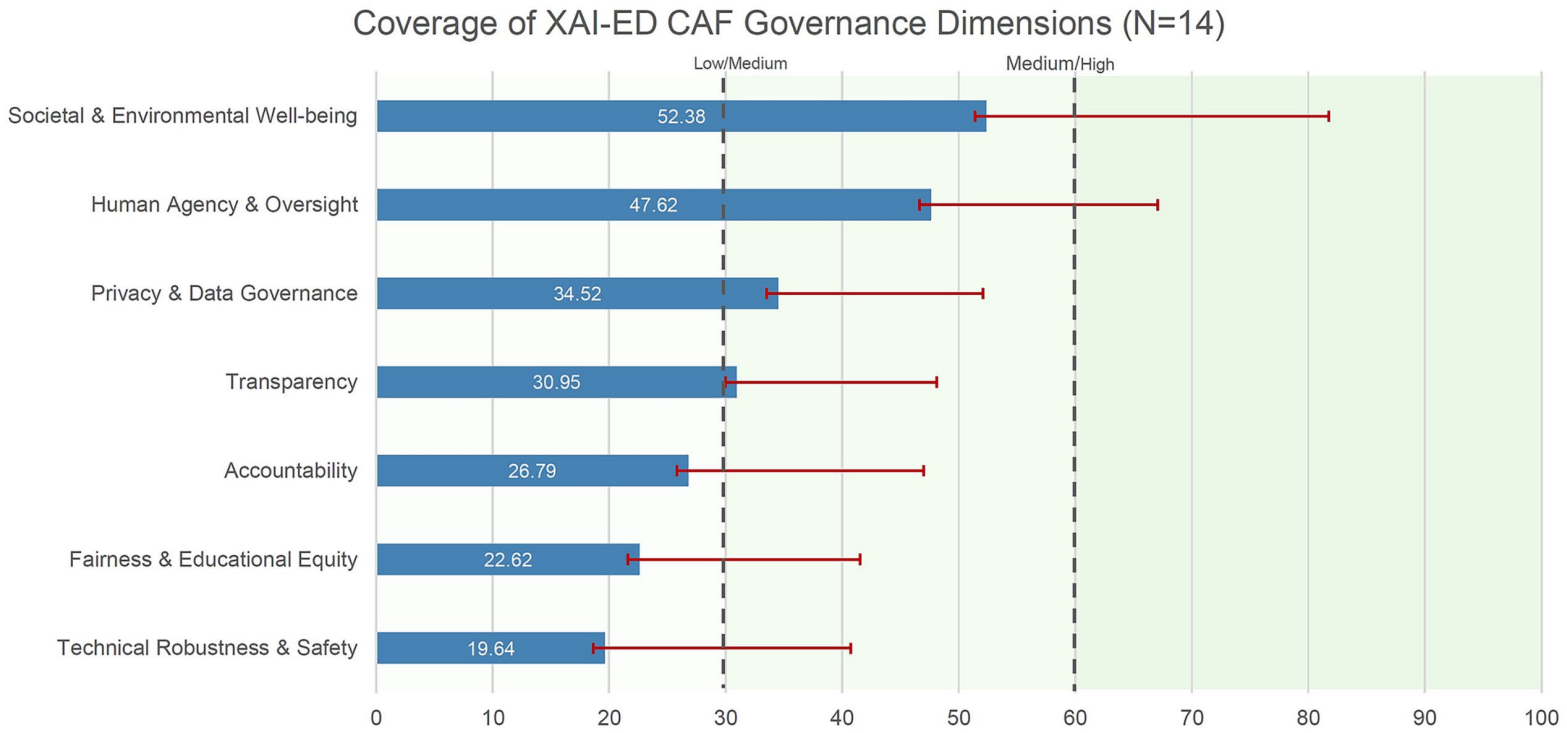
Coverage of XAI-ED CAF governance dimensions across Italian universities (*N* = 14). Horizontal bars show mean scores on a 0–100 scale for each ALTAI-based dimension adapted to an educational evaluation framework. Error bars indicate ±1 standard deviation. Scores were normalized using (mean item score ÷ 3) × 100, with item scores ranging from 0 (no evidence) to 3 (documented implementation with verifiable evidence). Vertical dashed lines at 30 and 60 indicate coverage thresholds. Dimensions are ordered by increasing mean score. Results suggest stronger emphasis on high-level ethical and societal commitments than on operational safeguards such as technical robustness and fairness, and considerable variability across institutions.

Scores normalized to 0–100 scale using formula: (mean item score ÷ 3) × 100, where items are scored 0–3. Coverage levels based on thresholds: high >60, medium 30–60, low <30. Dimensions ranked by mean score (highest to lowest). Comprehensive statistics including median values facilitate interpretation of distributional properties complemented by visual representation in Figure 1.

Figure 1 reveals two critical patterns. First, a clear hierarchy emerges with “soft governance” dimensions (addressing mission, oversight, and societal impact) showing substantially higher documentation than “hard governance” dimensions (requiring technical validation, bias monitoring, and fairness auditing). Second, the large standard deviations across all dimensions (ranging from SD = 17.12 to SD = 29.38) indicate substantial institutional heterogeneity, with no evidence of convergence toward shared governance models among early adopters.

**Figure 2:**
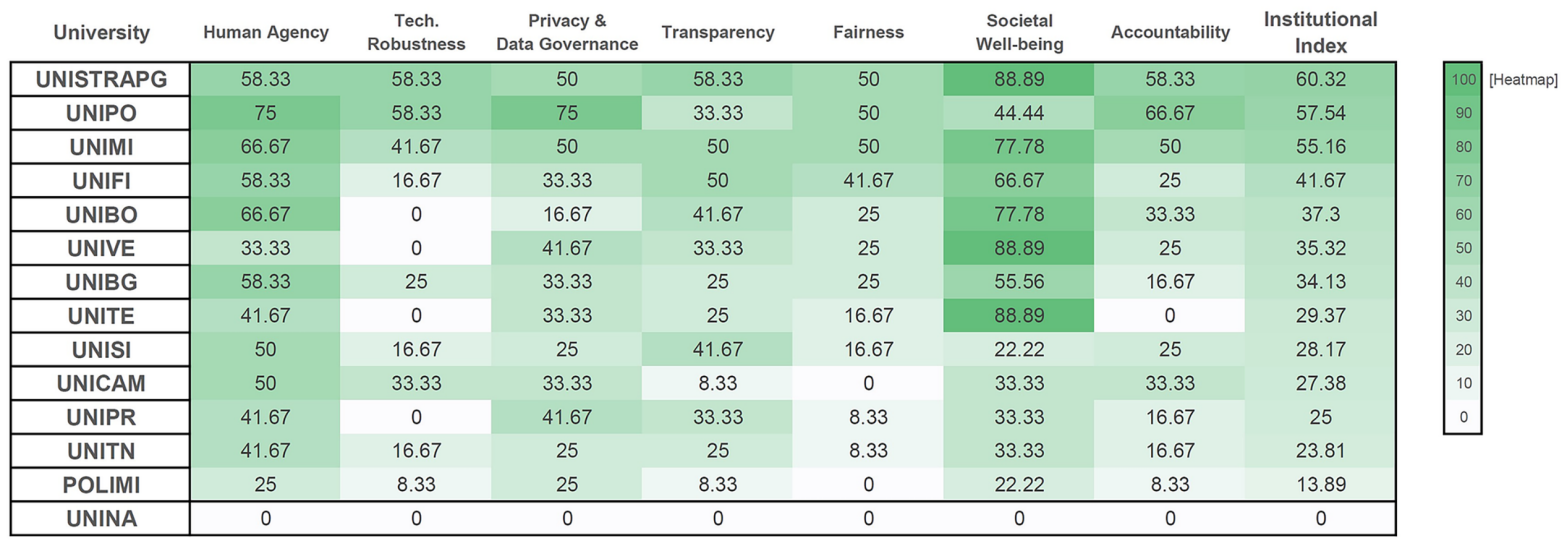
Institutional-level scores across XAI-ED CAF governance dimensions. Scores normalized to 0–100 scale: (mean item score ÷ 3) × 100. Items scored 0–3 (0 = no evidence, 1 = weak, 2 = partial, 3 = documented implementation). Institutional Index = unweighted mean of seven dimensions. UNINA (baseline case) scored zero, confirming instrument sensitivity. Higher values reflect stronger documented alignment with the framework’s criteria across dimensions. Governance maturity levels: nascent (<35), developing (35–50), established (>50).

Institutional Index scores ranged from 0.00 to 60.32 (M = 33.66, SD = 17.89), revealing substantial governance heterogeneity among early-adopting institutions (Figure 2). Applying threshold-based interpretation to facilitate comparative assessment, institutions cluster into three governance maturity levels: nascent governance (scores <35: 8 institutions, including the baseline case), developing governance (scores 35–50: 3 institutions), and established governance (scores >50: 3 institutions).

The heatmap visualization in Figure 2 reveals three critical patterns. First, substantial inter-institutional variation exists even among early adopters, with Institutional Index scores spanning a 60-point range (UNINA: 0.00 to UNISTRAPG: 60.32). Second, Technical Robustness and Safety (second column) shows predominantly light shading across institutions, indicating systematic weakness in technical validation documentation regardless of overall governance maturity. Third, no institution achieves consistently high coverage across all dimensions: even high-performing institutions (UNIPO, UNISTRAPG, UNIMI) show uneven profiles with gaps in specific areas, suggesting selective rather than comprehensive governance implementation.

Even institutions in the established category reached only approximately 60% of maximum possible documentation, indicating that comprehensive governance across all seven ALTAI dimensions remains challenging for early adopters. No institution achieved scores above 90 in any single dimension, with maximum scores ranging from 50.00 (Fairness and Educational Equity) to 88.89 (Societal and Environmental Well-being). The concentration of scores in the nascent and developing categories (11 of 14 institutions below 50 points) suggests that most early-adopting universities have formalized governance frameworks in only selected areas, prioritizing aspirational commitments and compliance-oriented dimensions while leaving technical robustness and fairness monitoring substantially underdeveloped.

Distribution analysis identified systematic patterns. Technical Robustness and Safety showed median score of 16.67, with 50% of institutions scoring below 8.33. Zero minimum values across all dimensions confirmed instrument sensitivity to policy absence, while maximum scores below 90 across most dimensions indicated that comprehensive documentation remains challenging even for early adopters.

Qualitative review of coded evidence (complete item-level scores, consensus ratings, and qualitative notes available in Supplementary Materials 3) revealed four distinct documentation patterns among the 14 institutions analyzed:

- Seven institutions (UNITE, UNIBO, POLIMI, UNICAM, UNITN, UNIVE, and partially UNIPR) exhibited aspirational statements referencing ethical principles and GDPR compliance without operational mechanisms: notes consistently documented absence of structured procedures for validity testing, internal audits, appeal mechanisms, or specific governance roles for AI-supported assessment.
- Four institutions (UNIBG, UNIMI, UNIFI, UNISI) showed integration of existing frameworks through references to EU ethical codes and involvement of Data Protection Officers, yet connections to assessment remained indirect or implicit, with limited implementation details.
- Two institutions (UNIPO, UNISTRAPG) implemented purpose-built governance frameworks featuring explicit requirements for validity and reliability checks (Item 2.1: scores 2–3), lifecycle procedures (Item 2.3: scores 2–3), internal audit provisions (Item 2.4: scores 2), and bias monitoring protocols (Item 5.2: UNISTRAPG score 3).
- One institution (UNINA) lacked any AI policy documentation, scoring zero across all 27 items and thus confirming the instrument’s discriminant validity.

Cross-institutional patterns documented through consensus notes revealed that 12 of 13 institutions with policies failed to establish appeal procedures for AI-influenced assessment decisions (Item 1.2), articulate student rights to data access, rectification, or erasure in assessment contexts (Item 3.3), or mandate stakeholder consultation mechanisms (Item 5.4: 12 institutions scored 0). Where staff training provisions existed, qualitative review indicated they were neither mandatory nor specific to assessment oversight responsibilities.

## 5 Discussion

### 5.1 Instrument validation and methodological implications (RQ1)

The reliability coefficients indicate that outcome-focused governance assessment through document analysis is feasible but reveals structural tensions requiring acknowledgment. Krippendorff’s *α* = 0.838 exceeds conventional thresholds, while Fleiss’s *κ* = 0.626 indicates substantial but imperfect agreement. This discrepancy is not methodological artifact but reflects a substantive governance challenge: Italian university policies frequently employ aspirational language without specifying operational procedures, creating legitimate interpretive variation even among trained coders.

The lower reliability for intermediate scores reflects this structural limitation. Universities document governance intentions more readily than implementation mechanisms, a pattern with direct implications for regulatory compliance under the AI Act. This finding is not merely methodological but substantive, showing that institutions can articulate what they aspire to accomplish but struggle to specify how they will accomplish it or how they will verify accomplishment.

The substantial variation in pairwise agreement (κ: 0.438–0.922) suggests complications for the assumption that governance assessment can be disciplinarily neutral. The high agreement between technical specialists and lower agreement between governance and technical perspectives suggests that effective governance assessment requires interdisciplinary collaboration rather than single-perspective evaluation. This connects to Peterson and Broersen’s (2024) argument that AI systems cannot autonomously provide normative justifications. Applied to governance assessment, no checklist can eliminate interpretive variation rooted in different professional epistemologies. The XAI-ED CAF reduces but does not eliminate this variation.

The framework’s focus on policy documentation introduces a critical interpretive constraint: high scores indicate formalized governance frameworks but do not verify implementation effectiveness. This gap reflects substantive governance complexity rather than methodological artifact. Institutions may document comprehensive frameworks that remain unimplemented, implement effective informal mechanisms that remain undocumented, or defer AI adoption pending regulatory clarity. Document analysis cannot systematically distinguish among these scenarios.

The XAI-ED CAF measures institutional transparency about governance intentions (i.e., the willingness and capacity to formalize, articulate, and publicly commit to accountability standards) a legitimate but bounded dimension. For regulatory compliance, documented frameworks provide necessary evidence of accountability structures. However, compliance verification requires complementary methods: stakeholder interviews, technical audits verifying robustness claims, and outcome analysis assessing whether commitments translate into equitable results.

### 5.2 Governance patterns and institutional capacity (RQ2)

The dimension hierarchy observed among early-adopting institutions (Societal Well-being: M = 52.38 versus Technical Robustness: M = 19.64) reveals systematic misalignment between regulatory expectations and institutional capacity. The EU AI Act Annex III, point 3b classification implies technical safeguards comparable to medical devices or critical infrastructure. Yet universities examined lack organizational structures for algorithmic accountability at this level.

This gap reflects structural misalignment between regulatory assumptions and organizational reality rather than institutional negligence. Educational institutions possess expertise in pedagogical validity and learning assessment but not algorithmic auditing or technical system validation.

This raises questions about AI governance scholarship’s premise that sector-specific frameworks solve abstract ethical principles (Yan et al., 2025). The XAI-ED CAF reinterprets ALTAI through evaluation theory, yet empirical evidence suggests theoretical adaptation alone may be insufficient. Universities prioritize Societal Well-being and Human Agency because these align with existing institutional logics (strategic planning, quality assurance), while Technical Robustness and Fairness require capacities they lack and cannot develop without substantial resource investment.

The 13% adoption rate and institutional heterogeneity (Index: 0.00–60.32) indicate early-stage experimentation in Italian higher education. This finding carries two interpretive implications.

First, the small proportion of policy-adopting institutions limits claims about sector-wide governance patterns; observed practices may reflect institutional characteristics (resources, expertise, regulatory awareness) that distinguish early adopters from the broader population. Second, the analysis captures governance at a specific developmental moment (nearly one year before full AI Act applicability) when experimentation rather than standardization characterizes institutional responses.

Whether early-stage heterogeneity represents a transitional phase preceding convergence or reflects stable fragmentation cannot be determined from cross-sectional data. However, the absence of institutional convergence approaching the compliance deadline raises questions about the effectiveness of compliance-driven governance models unaccompanied by sector-specific implementation guidance. The governance patterns documented here should therefore be interpreted as exploratory evidence from policy pioneers rather than representative characteristics of Italian higher education’s response to AI regulation.

Three institutions exceeded 50 points on the Institutional Index, suggesting comprehensive documentation is achievable in early adoption phases. Qualitative analysis identified common features: designated AI governance committees, explicit references to EU regulatory frameworks, and cross-functional policy development involving legal, technical, and pedagogical expertise. These features are resource-intensive and may not scale across institutions with different capacities.

The baseline comparison confirms framework sensitivity but highlights a critical limitation. Policy absence does not necessarily indicate governance absence: institutions may implement informal accountability mechanisms, rely on existing quality assurance structures adapted for AI contexts, or defer AI adoption pending regulatory clarity. Conversely, policy presence does not guarantee effective implementation: comprehensive documentation may coexist with weak operational capacity. Document analysis cannot distinguish between these scenarios. The framework measures institutional transparency about governance intentions, which is legitimate but bounded.

Cross-national comparative evidence contextualizes these findings within broader governance stratification patterns. Italy’s 13% adoption rate aligns with European surveys showing 80% of institutions lacking policies (Media and Learning Association, 2023) and UNESCO’s (2025) global finding of 19% formal adoption. However, Kontogiannis et al.’ (2024) analysis of the world’s top 50 universities found 82% had publicly accessible GenAI guidelines, substantially higher than comprehensive national systems. This disparity suggests governance adoption is resource-stratified: elite, research-intensive institutions with greater technical capacity, legal expertise, and administrative infrastructure implement frameworks earlier and more comprehensively than institutions with limited resources. The 13 Italian institutions analyzed here likely represent the resource-advantaged segment of the national system (comparable to internationally elite institutions in capacity though not in resources) while the broader system lags substantially. Stracke et al.’ (2025) analysis of 15 European policies identified heterogeneity and framework gaps comparable to patterns documented here, reinforcing that Italian universities’ challenges reflect systemic European conditions rather than national anomalies. However, whether resource-stratified adoption persists as regulatory deadlines approach, or whether late adopters develop alternative governance models adapted to resource constraints, requires longitudinal investigation.

### 5.3 Theoretical integration and its empirical complications

The theoretical integration of ALTAI with educational evaluation frameworks proves conceptually coherent but empirically demanding. Messick’s (1995) consequential validity requires evidence of construct validity and fairness monitoring, yet low Technical Robustness scores (M = 19.64) indicate universities struggle to produce such evidence. Kirkpatrick’s (2006) four-level model structures evaluation across reaction, learning, behavior, and results, but early-adopting universities document Level 1 reactions (aspirational statements) far more readily than Level 3–4 outcomes (systematic evidence of fair results). Stufflebeam’s (1983) CIPP framework addresses inputs, processes, and products; moderate Privacy scores (M = 34.52) suggest partial capacity, while low Accountability scores (M = 26.79) indicate underdeveloped outcome evaluation.

These patterns reveal a critical gap: universities can articulate governance intentions using evaluation frameworks but struggle to demonstrate systematic outcome assessment. Whether this reflects early-stage implementation or structural incompatibility between evaluation theory and algorithmic systems requires longitudinal investigation.

The framework demonstrates that outcome-focused governance requires capacities distinct from compliance-oriented approaches. Regulatory frameworks emphasizing ex-ante conformity (system documentation, risk assessments) differ fundamentally from evaluation frameworks requiring ex-post evidence (validity studies, equity audits, impact assessments).

### 5.4 Societal implications and regulatory laboratories

The findings carry implications for educational equity and institutional accountability. Without systematic outcome evaluation, AI-mediated assessment decisions affecting student progression and certification may escape meaningful oversight. Vulnerable student populations may consequently face algorithmically mediated evaluations lacking the validity and fairness safeguards regulatory frameworks presume exist.

This concern becomes particularly acute given the governance patterns observed. The prioritization of aspirational statements over operational mechanisms means institutional commitments to fairness and transparency may not translate into practices protecting student rights. The low documentation of Technical Robustness and Fairness dimensions suggests early-adopting institutions lack capacity to demonstrate that AI-mediated assessments produce valid and equitable results.

However, the study also reveals universities functioning as regulatory laboratories. The 13 institutions with AI policies represent experimental governance approaches testing how abstract regulatory obligations translate into sector-specific practices. This experimentation may be valuable because it reveals implementation challenges regulatory frameworks did not anticipate. The governance deficit documented here suggests regulatory frameworks must account for organizational realities and provide implementation guidance rather than assume abstract requirements translate automatically into practice.

This positions universities as both regulated entities subject to compliance obligations and sites of governance innovation developing sector-specific accountability models. The XAI-ED CAF contributes by providing structured self-assessment tools while revealing where theoretical frameworks encounter practical limitations.

## 6 Limitations and future research directions

Four limitations constrain interpretation of findings. First, document analysis assesses policy articulation rather than implementation quality, measuring whether institutions can formalize governance intentions but not whether formalized intentions translate into practice. High scores may reflect documentation capacity rather than governance effectiveness. Validation of this relationship requires complementary methods integrating stakeholder interviews and implementation case studies.

Second, the exploratory sample of early-adopting institutions substantially limits generalizability. While the 13% adoption rate is a population-level finding, governance patterns reflect only institutions that had formalized AI policies by September 2025 (approximately one in eight Italian universities). These early adopters may systematically differ from the broader population in technical capacity, regulatory awareness, resource availability, or institutional culture, with documentation patterns likely representing best-case scenarios rather than typical practices.

Cross-sectional comparison of early versus late adopters and longitudinal tracking of governance evolution are necessary to distinguish early-adoption artifacts from systemic patterns. Until such comparative data exist, findings should be interpreted as exploratory evidence from policy pioneers rather than representative sector-wide governance characteristics.

Third, while the Italian context may reflect national specificities (regulatory culture, institutional autonomy traditions, ministerial guidance patterns), available comparative evidence suggests Italian governance patterns align with broader European and global trends rather than representing national anomalies. Italy’s 13% adoption rate is consistent with the Media and Learning Association’s (2023) finding that 80% of European institutions lacked AI policies and with UNESCO’s (2025) global adoption rate of 19%. Stracke et al.’ (2025) analysis of 15 policies across eight European countries revealed heterogeneity in governance approaches and framework gaps comparable to patterns documented here. However, governance adoption appears resource-stratified globally: Kontogiannis et al.’ (2024) analysis found 82% of the world’s top 50 universities had GenAI guidelines, substantially higher than comprehensive national systems. The 13 Italian institutions analyzed here may represent the resource-advantaged segment of the national system (possessing greater technical capacity, legal expertise, and administrative infrastructure than the broader population) limiting generalizability to institutions with fewer resources. Nevertheless, systematic cross-national comparative studies tracking policy evolution across institutions with varying resource levels, regulatory contexts, and governance maturity remain necessary to distinguish resource-driven patterns from context-specific adaptations and to determine whether late adopters develop alternative governance models suited to resource-constrained environments.

Fourth, the framework requires validation beyond exploratory reliability testing. Acceptable Krippendorff’s *α* establishes proof-of-concept feasibility but does not confirm construct validity (whether items measure intended dimensions), criterion validity (whether scores predict implementation quality), or predictive validity (whether early governance predicts long-term compliance success). Construct validation through expert Delphi panels represents a possible next step, followed by criterion validation linking documented governance to measured outcomes.

Future research could pursue three directions. Longitudinal monitoring of Italian universities approaching the 2026 deadline would document whether early patterns predict compliance outcomes and whether late adopters exhibit different governance approaches. Cross-national comparative studies would clarify whether identified patterns reflect Italian specificities or European governance challenges. Multi-method validation integrating document analysis with implementation assessment and stakeholder evaluation would examine whether the framework adequately captures governance quality or requires substantial refinement.

## 7 Conclusion

This study examined whether ALTAI’s trustworthy AI requirements could be operationalized for institutional self-assessment of AI-based learning outcome assessment through educational evaluation theory. Three contributions emerge with implications extending beyond methodological validation.

First, the empirical evidence shows outcome-focused governance can be assessed systematically through document analysis but reveals critical gaps between regulatory expectations and institutional capacity among early-adopting universities. Italian universities document aspirational commitments more readily than operational mechanisms, with Technical Robustness and Fairness showing particularly weak implementation. This pattern reflects structural misalignment: the EU AI Act presumes technical validation capacities educational institutions do not routinely possess. This finding suggests that sector-specific standards must account for organizational realities and provide implementation guidance.

Second, the theoretical integration of ALTAI with evaluation theory proves conceptually coherent but empirically demanding in early governance development phases. Universities can articulate governance using evaluative vocabulary but struggle to produce systematic outcome evidence. This finding raises questions about assumptions that framework adaptation solves governance implementation problems. The difficulty lies not in conceptual translation but in operational gaps between documenting intentions and demonstrating results. This distinction matters for understanding what governance frameworks accomplish: they provide structured accountability language but do not substitute for technical and organizational capacities required to generate validity evidence, conduct fairness audits, or assess consequential impacts. Cross-national evidence reinforces this finding: while 82% of elite global universities have developed GenAI guidelines (Kontogiannis et al., 2024), only 13–19% of comprehensive national systems have done so (Media and Learning Association, 2023; UNESCO, 2025), suggesting governance capacity is resource-stratified and that evaluation frameworks developed for well-resourced contexts may not translate directly to institutions with limited technical and administrative capacities.

Third, the 13% adoption rate among Italian universities approaching the August 2026 compliance deadline indicates voluntary governance development remains limited. Substantial institutional heterogeneity among early adopters (Index: 0.00–60.32) suggests experimentation rather than convergence toward shared models. Whether coordination emerges through regulatory pressure, professional networking, or ministerial guidance remains uncertain. However, baseline evidence shows most Italian institutions lack documented AI governance frameworks, creating compliance risks that may require policy intervention including sector-specific guidance and capacity-building support.

The methodological contribution lies in showing that exploratory validation can establish proof-of-concept feasibility while identifying areas requiring refinement. Acceptable reliability coefficients (Krippendorff’s *α* = 0.838) support cautious framework adoption for policy document analysis in governance contexts, while moderate Fleiss’s *κ* (0.626) and low agreement on intermediate scores indicate clearer operational definitions are necessary. The finding that disciplinary background influences policy interpretation (pairwise κ: 0.438–0.922) suggests effective governance assessment requires interdisciplinary collaboration rather than single-perspective evaluation.

The implications concern educational equity and institutional accountability. If universities implement AI-mediated assessment without systematic outcome evaluation, algorithmic decisions affecting student progression and certification may escape meaningful oversight. Vulnerable student populations face particular risks when algorithmically mediated evaluations lack validity and fairness safeguards. The framework provides tools for demonstrating accountability, but empirical evidence shows that early-adopting universities and, by extension, most institutions have not yet developed such capabilities. A potential consequence is that students may face algorithmically mediated evaluations lacking the safeguards regulatory frameworks presume exist.

These findings suggest three concrete directions for bridging the gap between regulatory requirements and institutional capacity. First, the Ministry of Universities and Research should develop sector-specific guidance translating EU AI Act obligations into operational procedures tailored to educational assessment contexts. Such guidance should specify minimum documentation standards, provide templates for validity testing protocols, and clarify how existing quality assurance mechanisms can be adapted for AI governance.

The observed heterogeneity in governance approaches (institutional index: 0.00–60.32) indicates that institutions lack shared reference models; ministerial guidance would reduce fragmentation and support convergence toward evidence-based practices.

Second, universities require capacity-building support addressing the technical-pedagogical competency gap documented in this study. The low scores on Technical Robustness and Safety (M = 19.64) and Fairness monitoring (M = 22.62) reflect structural limitations rather than institutional negligence. Effective interventions might include: (a) development of inter-university consortia pooling technical expertise for shared AI governance infrastructure; (b) integration of AI accountability modules into faculty development programs, particularly for assessment design and learning analytics roles; (c) establishment of regional AI governance support centers providing consultation on validity testing, bias auditing, and stakeholder impact assessment. These mechanisms would enable institutions to move from aspirational commitments to operational accountability.

Third, standardized self-assessment procedures should be integrated into existing quality assurance frameworks. The XAI-ED CAF demonstrates that outcome-focused governance can be systematically assessed through document analysis, but current accreditation processes do not routinely examine AI governance in educational assessment. National quality assurance agencies (ANVUR in Italy, or equivalent bodies in other EU member states) could incorporate AI governance indicators into periodic institutional reviews, creating accountability mechanisms while respecting institutional autonomy. This approach would leverage existing evaluation infrastructure rather than imposing parallel compliance bureaucracies, aligning with the regulatory laboratory model where universities develop context-appropriate governance practices within structured accountability frameworks.

While these recommendations emerge from systematic analysis of Italian early-adopting institutions, their implementation must acknowledge the research boundaries documented here. Document analysis measures policy articulation rather than implementation quality, findings on governance patterns reflect early adopters and may not generalize to later-adopting institutions, the Italian context limits cross-national generalizability, and the framework requires validation beyond exploratory reliability testing. Construct validity, criterion validity, and predictive validity remain unestablished. These are not correctable deficiencies but scope limitations defining what the study shows and what remains unknown.

The study indicates that AI governance in education remains at an early stage characterized by regulatory uncertainty, institutional experimentation, and limited shared models. The XAI-ED CAF shows that outcome-focused governance grounded in educational evaluation theory can be operationalized, but empirical evidence reveals the complexity of bridging regulatory compliance with pedagogical accountability particularly in early governance development phases. Whether the framework proves useful depends on subsequent validation, broader application across institutions at different governance maturity levels, and institutional capacity development addressing the structural gaps this research has identified.

## Data Availability

The original contributions presented in the study are included in the article/Supplementary material, further inquiries can be directed to the corresponding author.
