# Supplementary material for: Testing the applicability of a governance checklist for high-risk AI-based learning outcome assessment in Italian universities under the EU AI act annex III
Source: Front Artif Intell. 2025 Dec 11;8:1718613. doi: 10.3389/frai.2025.1718613 (PMC12738910; doi:10.3389/frai.2025.1718613)
Supplement: Supplementary file 1 [file Data_Sheet_1.pdf]

Supplementary Material

1 Complete dataset generated during the screening phase, covering all 100 universities.

id	university_name	region	city	website_url	geographical_distribution	institutional_size	legal_status	availability_of_AI_related_policies	URL	last_update
1	Università degli Studi “Gabriele d’Annunzio” di Chieti-Pescara	Abruzzo	Chieti-Pescara	https://www.unich.it/	South/Islands	Medium (10–40k)	Public	No		
2	Università degli Studi dell’Aquila	Abruzzo	L’Aquila	https://www.univaq.it	South/Islands	Medium (10–40k)	Public	No		
3	Università degli Studi di Teramo	Abruzzo	Teramo	https://www.unite.it	South/Islands	Small (<10k)	Public	Yes	https://www.unite.it/UniTE/Giurisprudenza/Linee_di_indirizzo_Universita_di_Tera	2025-03

id	university_name	region	city	website_url	geographical_distribution	institutional_size	legal_status	availability_of_AI_related_policies	URL	last_update
									mo_per_un_impiego_etico_consapevole_e_responsabile_dell_Intelligenza_Artificiale_generativa	
4	Università degli Studi della Basilicata	Basilicata	Potenza	https://portale.unibas.it	South/Islands	Small (<10k)	Public	No		
5	Università degli Studi “Magna Græcia” di Catanzaro	Calabria	Catanzaro	https://www.umg.it	South/Islands	Medium (10–40k)	Public	No		
6	Università della Calabria	Calabria	Rende (CS)	https://www.unical.it	South/Islands	Medium (10–40k)	Public	No		

id	university_name	region	city	website_url	geographical_distribution	institutional_size	legal_status	availability_of_AI_related_policies	URL	last_update
7	Università degli Studi "Mediterranea" di Reggio Calabria	Calabria	Reggio Calabria	https://www.unirc.it	South/Islands	Small (<10k)	Public	No		
8	Università degli Studi di Napoli "Federico II"	Campania	Napoli	https://www.unina.it	South/Islands	Large (>40k)	Public	No		
9	Università degli Studi di Napoli "L'Orientale"	Campania	Napoli	https://www.unior.it	South/Islands	Medium (10–40k)	Public	No		
10	Università degli Studi di Napoli "Parthenope"	Campania	Napoli	https://www.uniparthenope.it	South/Islands	Medium (10–40k)	Public	No		

id	university_name	region	city	website_url	geographical_distribution	institutional_size	legal_status	availability_of_AI_related_policies	URL	last_update
11	Università degli Studi del Sannio	Campania	Benevento	https://www.unisannio.it	South/Islands	Small (<10k)	Public	No		
12	Università degli Studi della Campania “Luigi Vanvitelli”	Campania	Caserta	https://www.unicampania.it	South/Islands	Medium (10–40k)	Public	No		
13	Università degli Studi di Salerno	Campania	Fisciano (SA)	https://www.unisa.it	South/Islands	Medium (10–40k)	Public	No		
14	Alma Mater Studiorum - Università di Bologna	Emilia-Romagna	Bologna	https://www.unibo.it	North	Large (>40k)	Public	Yes	https://www.unibo.it/it/ateneo/statuto-norme-strategie-bilanci/intelligenza-artificiale	2025-01

id	university_name	region	city	website_url	geographical_distribution	institutional_size	legal_status	availability_of_AI_related_policies	URL	last_update
15	Università degli Studi di Ferrara	Emilia-Romagna	Ferrara	https://www.unife.it	North	Medium (10–40k)	Public	No		
16	Università degli Studi di Modena e Reggio Emilia	Emilia-Romagna	Modena	https://www.unimore.it	North	Medium (10–40k)	Public	No		
17	Università degli Studi di Parma	Emilia-Romagna	Parma	https://www.unipr.it	North	Medium (10–40k)	Public	Yes	https://www.unipr.it/linee-guida-intelligenza-artificiale	2025-05
18	Università degli Studi di Trieste	Friuli-Venezia Giulia	Trieste	https://www.units.it	North	Medium (10–40k)	Public	No		
19	Università degli Studi di Udine	Friuli-Venezia Giulia	Udine	https://www.uniud.it	North	Medium (10–40k)	Public	No		

id	university_name	region	city	website_url	geographical_distribution	institutional_size	legal_status	availability_of_AI_related_policies	URL	last_update
20	Università degli Studi di Cassino e del Lazio Meridionale	Lazio	Cassino (FR)	https://www.unicas.it	Center	Small (<10k)	Public	No		
21	Università degli Studi di Roma “Foro Italico”	Lazio	Roma	https://www.uniroma4.it	Center	Small (<10k)	Public	No		
22	Università degli Studi di Roma “La Sapienza”	Lazio	Roma	https://www.uniroma1.it	Center	Large (>40k)	Public	No		
23	Università degli Studi di Roma “Tor Vergata”	Lazio	Roma	https://web.uniroma2.it	Center	Medium (10–40k)	Public	No		

id	university_name	region	city	website_url	geographical_distribution	institutional_size	legal_status	availability_of_AI_related_policies	URL	last_update
24	Università degli Studi di Roma Tre	Lazio	Roma	https://www.uniroma3.it	Center	Medium (10–40k)	Public	No		
25	Università degli Studi della Tuscia	Lazio	Viterbo	https://www.unitus.it	Center	Small (<10k)	Public	No		
26	Università degli Studi di Genova	Liguria	Genova	https://unige.it	North	Medium (10–40k)	Public	No		
27	Università degli Studi di Bergamo	Lombardia	Bergamo	https://www.unibg.it	North	Medium (10–40k)	Public	Yes	https://www.unibg.it/ateneo/amministrazione/linee-guida-intelligenza-artificiale	2025-06

id	university_name	region	city	website_url	geographical_distribution	institutional_size	legal_status	availability_of_AI_related_policies	URL	last_update
28	Università degli Studi di Brescia	Lombardia	Brescia	https://www.unibs.it	North	Medium (10–40k)	Public	No		
29	Università degli Studi dell'Insubria	Lombardia	Varese	https://www.uninsubria.it	North	Medium (10–40k)	Public	No		
30	Università degli Studi di Milano	Lombardia	Milano	https://www.unimi.it	North	Large (>40k)	Public	Yes	https://www.unimi.it/it/ateneo/normative/linee-guida/decalogo-un-utilizzo-etico-legittimo-e-consapevole-di-strumenti-dintelligenza-artificiale-ai-tutte	2024-12

id	university_name	region	city	website_url	geographical_distribution	institutional_size	legal_status	availability_of_AI_related_policies	URL	last_update
31	Università degli Studi di Milano-Bicocca	Lombardia	Milano	https://www.unimib.it	North	Medium (10–40k)	Public	No		
32	Università degli Studi di Pavia	Lombardia	Pavia	https://www.unipv.it	North	Medium (10–40k)	Public	No		
33	Politecnico di Milano	Lombardia	Milano	https://www.polimi.it	North	Large (>40k)	Public	Yes	https://www.normativa.polimi.it/fileadmin/user_upload/regolamenti/privacy_e_sicurezza/REGOLAMENTO_trattamento_dati_e_ICT__marzo2025.pdf?utm_source=chatgpt.com	2025-03

id	university_name	region	city	website_url	geographical_distribution	institutional_size	legal_status	availability_of_AI_related_policies	URL	last_update
34	Università Politecnica delle Marche	Marche	Ancona	https://www.univpm.it	Center	Medium (10–40k)	Public	No		
35	Università degli Studi di Camerino	Marche	Camerino	https://www.unicam.it	Center	Small (<10k)	Public	Yes	https://www.unicam.it/sites/default/files/documenti-pag/2024/DR_Linee%20guida%20IA_signed.pdf	2024-07
36	Università degli Studi di Macerata	Marche	Macerata	https://www.unimc.it	Center	Small (<10k)	Public	No		
37	Università degli Studi di Urbino “Carlo Bo”	Marche	Urbino	https://www.uniurb.it	Center	Medium (10–40k)	Public	No		

id	university_name	region	city	website_url	geographical_distribution	institutional_size	legal_status	availability_of_AI_related_policies	URL	last_update
38	Università degli Studi del Molise	Molise	Campobasso	https://www.unimol.it	Center	Small (<10k)	Public	No		
39	Università degli Studi del Piemonte Orientale “Amedeo Avogadro”	Piemonte	Vercelli	https://www.uniupo.it	North	Medium (10–40k)	Public	Yes	https://www.uniupo.it/it/ateneo/regole-trasparenza-sindacati/normativa/linee-guida-transitorie-utilizzo-strumenti-di-intelligenza-artificiale	2025-02
40	Università degli Studi di Torino	Piemonte	Torino	https://www.unito.it	North	Large (>40k)	Public	No		
41	Politecnico di Torino	Piemonte	Torino	https://www.polito.it	North	Medium (10–40k)	Public	No		

id	university_name	region	city	website_url	geographical_distribution	institutional_size	legal_status	availability_of_AI_related_policies	URL	last_update
42	Politecnico di Bari	Puglia	Bari	https://www.poliba.it	South/Islands	Medium (10–40k)	Public	No		
43	Università degli Studi di Bari “Aldo Moro”	Puglia	Bari	https://www.uniba.it	South/Islands	Large (>40k)	Public	No		
44	Università degli Studi di Foggia	Puglia	Foggia	https://www.unifg.it	South/Islands	Medium (10–40k)	Public	No		
45	Università del Salento	Puglia	Lecce	https://www.unisalento.it	South/Islands	Medium (10–40k)	Public	No		
46	Università degli Studi di Cagliari	Sardegna	Cagliari	https://www.unica.it	South/Islands	Medium (10–40k)	Public	No		
47	Università degli Studi di Sassari	Sardegna	Sassari	https://www.uniss.it	South/Islands	Medium (10–40k)	Public	No		

id	university_name	region	city	website_url	geographical_distribution	institutional_size	legal_status	availability_of_AI_related_policies	URL	last_update
48	Università degli Studi di Catania	Sicilia	Catania	https://www.unict.it	South/Islands	Medium (10–40k)	Public	No		
49	Università degli Studi di Messina	Sicilia	Messina	https://www.unime.it	South/Islands	Medium (10–40k)	Public	No		
50	Università degli Studi di Palermo	Sicilia	Palermo	https://www.unipa.it	South/Islands	Large (>40k)	Public	No		
51	Università degli Studi di Firenze	Toscana	Firenze	https://www.unifi.it	Center	Large (>40k)	Public	Yes	https://www.unifi.it/it/node/9535	2025-03
52	Università degli Studi di Pisa	Toscana	Pisa	https://www.unipi.it	Center	Large (>40k)	Public	No		
53	Università degli Studi di Siena	Toscana	Siena	https://www.unisi.it	Center	Medium (10–40k)	Public	Yes	https://www.unisi.it/sites/default/files/albo_pretorio/allegati/Linee%20guida	2023-09

id	university_name	region	city	website_url	geographical_distribution	institutional_size	legal_status	availability_of_AI_related_policies	URL	last_update
									%20UNISI%20Chat%20GPT_.pdf	
54	Università per Stranieri di Siena	Toscana	Siena	https://www.unistrasi.it	Center	Small (<10k)	Public	No		
55	Università degli Studi di Trento	Trentino-Alto Adige	Trento	https://www.unitn.it	North	Medium (10–40k)	Public	Yes	https://www.unitn.it/sites/default/files/2025-03/W_054_25_Policy_IA_ver_03.pdf	2025-03
56	Università degli Studi di Perugia	Umbria	Perugia	https://www.unipg.it	Center	Medium (10–40k)	Public	No		

id	university_name	region	city	website_url	geographical_distribution	institutional_size	legal_status	availability_of_AI_related_policies	URL	last_update
57	Università per Stranieri di Perugia	Umbria	Perugia	https://www.unistrapg.it	Center	Small (<10k)	Public	Yes	https://www.unistrapg.it/sites/default/files/docs/statuto-regolamenti/linee_guida_ai.pdf	2025-04
58	Università Ca' Foscari Venezia	Veneto	Venezia	https://www.unive.it	North	Medium (10–40k)	Public	Yes	https://www.unive.it/pag/49804/	2025-09
59	Università Iuav di Venezia	Veneto	Venezia	https://www.iuav.it	North	Small (<10k)	Public	No		
60	Università degli Studi di Padova	Veneto	Padova	https://www.unipd.it	North	Large (>40k)	Public	No		
61	Università degli Studi di Verona	Veneto	Verona	https://www.univr.it	North	Medium (10–40k)	Public	No		

id	university_name	region	city	website_url	geographical_distribution	institutional_size	legal_status	availability_of_AI_related_policies	URL	last_update
62	Università della Valle d’Aosta / Université de la Vallée d’Aoste	Val d’Aosta	Aosta	https://www.univda.it/	North	Small (<10k)	Private	No		
63	Libera università di Bolzano / Freie Universität Bozen / Free University of Bozen-Bolzano	Trentino-Alto Adige	Bolzano	https://www.unibz.it/	North	Small (<10k)	Private	No		
64	Università degli Studi di Enna “Kore”	Sicilia	Enna	https://www.unikore.it/	South/Islands	Small (<10k)	Private	No		
65	Università degli Studi di Scienze	Piemonte	Bra	https://www.unisg.it/	North	Small (<10k)	Private	No		

id	university_name	region	city	website_url	geographical_distribution	institutional_size	legal_status	availability_of_AI_related_policies	URL	last_update
	Gastronomiche									
66	Libera Università Mediterranea Giuseppe Degennaro	Puglia	Casamassima	https://www.lum.it/	South/Islands	Small (<10k)	Private	No		
67	Università Carlo Cattaneo LIUC	Lombardia	Castellanza	https://www.liuc.it/	North	Small (<10k)	Private	No		
68	Libera università di lingue e comunicazione IULM	Lombardia	Milano	https://www.iulm.it/	North	Small (<10k)	Private	No		
69	Università Cattolica del Sacro Cuore	Lombardia	Milano	http://www.unicatt.it/	North	Large (>40k)	Private	No		

id	university_name	region	city	website_url	geographical_distribution	institutional_size	legal_status	availability_of_AI_related_policies	URL	last_update
70	Università commerciale Luigi Bocconi	Lombardia	Milano	https://unibocconi.it/	North	Medium (10–40k)	Private	No		
71	Università Vita-Salute San Raffaele	Lombardia	Milano	https://www.unisr.it/	North	Small (<10k)	Private	No		
72	Humanitas University	Lombardia	Pieve Emanuele	https://www.hunimed.eu/	North	Small (<10k)	Private	No		
73	Università degli Studi "Suor Orsola Benincasa"	Campania	Napoli	https://www.unisob.na.it/	South/Islands	Small (<10k)	Private	No		
74	Università per stranieri "Dante Alighieri"	Calabria	Reggio Calabria	http://www.unistrada.it/	South/Islands	Small (<10k)	Private	No		

id	university_name	region	city	website_url	geographical_distribution	institutional_size	legal_status	availability_of_AI_related_policies	URL	last_update
	di Reggio Calabria									
75	Luiss Guido Carli	Lazio	Roma	https://www.luiss.it/	Center	Medium (10–40k)	Private	No		
76	Libera Università Maria Santissima Assunta	Lazio	Roma	https://www.lumsa.it/	Center	Small (<10k)	Private	No		
77	Università Campus Bio-Medico	Lazio	Roma	https://www.unicampus.it/	Center	Small (<10k)	Private	No		
78	Università degli Studi Internazionali di Roma	Lazio	Roma	https://www.unint.eu/	Center	Small (<10k)	Private	No		
79	Link Campus University	Lazio	Roma	https://www.unilink.it/	Center	Small (<10k)	Private	No		

id	university_name	region	city	website_url	geographical_distribution	institutional_size	legal_status	availability_of_AI_related_policies	URL	last_update
80	Università Europea di Roma	Lazio	Roma	https://www.uer.it/	Center	Small (<10k)	Private	No		
81	Saint Camillus International University of Health Sciences	Lazio	Roma	https://unicamillus.org/	Center	Small (<10k)	Private	No		
82	Università telematica Giustino Fortunato	Campania	Benevento	https://www.unifortunato.eu/	South/Islands	Small (<10k)	Distance-learning	No		
83	Università telematica degli studi IUL	Toscana	Firenze	https://www.iuline.it/	Center	Small (<10k)	Distance-learning	No		
84	Università telematica "Pegaso"	Campania	Napoli	https://www.unipegaso.it/	South/Islands	Large (>40k)	Distance-learning	No		

id	university_name	region	city	website_url	geographical_distribution	institutional_size	legal_status	availability_of_AI_related_policies	URL	last_update
85	Università telematica eCampus	Lombardia	Novedrate	https://www.uniecampus.it/	North	Large (>40k)	Distance-learning	No		
86	Università telematica "Guglielmo Marconi"	Lazio	Roma	https://www.unimarconi.it/	Center	Medium (10–40k)	Distance-learning	No		
87	Università degli Studi "Niccolò Cusano"	Lazio	Roma	https://www.unicusano.it/	Center	Medium (10–40k)	Distance-learning	No		
88	Università telematica internazionale "UniNettuno"	Lazio	Roma	https://www.uninettunouniversity.net/	Center	Medium (10–40k)	Distance-learning	No		
89	Università telematica San Raffaele	Lazio	Roma	https://www.uniroma5.it/	Center	Small (<10k)	Distance-learning	No		

id	university_name	region	city	website_url	geographical_distribution	institutional_size	legal_status	availability_of_AI_related_policies	URL	last_update
90	Università telematica UNITELMA Sapienza	Lazio	Roma	https://www.unitelmasapienza.it/	Center	Small (<10k)	Distance-learning	No		
91	Università telematica Universitas Mercatorum	Lazio	Roma	https://www.unimercatorum.it/	Center	Large (>40k)	Distance-learning	No		
92	Università telematica “Leonardo da Vinci”	Abruzzo	Torrevecchia Teatina	https://unitelematicadavinci.ch/	South/Islands	Small (<10k)	Distance-learning	No		
93	Scuola universitaria superiore “Gran Sasso Science	Abruzzo	Aquila	https://gssi.it/	South/Islands	Small (<10k)	Special regulation	No		

id	university_name	region	city	website_url	geographical_distribution	institutional_size	legal_status	availability_of_AI_related_policies	URL	last_update
	Institute" (GSSI)									
94	Scuola Superiore Meridionale (SSM) - Napoli	Campania	Napoli	https://www.ssmeridionale.it/it-it/home	South/Islands	Small (<10k)	Special regulation	No		
95	Scuola Internazionale Superiore di Studi Avanzati (SISSA) - Trieste	Friuli-Venezia Giulia	Trieste	https://www.sissa.it/	North	Small (<10k)	Special regulation	No		
96	Centro Alti Studi per la Difesa (CASD) - Roma	Lazio	Roma	https://www.casd.it/	Center	Small (<10k)	Special regulation	No		
97	Istituto Universitario di Studi Superiori	Lombardia	Pavia	https://www.iusspavia.it/	North	Small (<10k)	Special regulation	No		

id	university_name	region	city	website_url	geographical_distribution	institutional_size	legal_status	availability_of_AI_related_policies	URL	last_update
	(IUSS) - Pavia									
98	Scuola IMT Alti Studi - Lucca	Toscana	Lucca	https://www.imtlucca.it/	Center	Small (<10k)	Special regulation	No		
99	Scuola Normale Superiore (SNS) - Pisa	Toscana	Pisa	https://www.sns.it/	Center	Small (<10k)	Special regulation	No		
100	Scuola superiore "Sant'Anna" - Pisa	Toscana	Pisa	https://www.santannapisa.it/it	Center	Small (<10k)	Special regulation	No		

2 XAI-ED CAF Checklist.

The checklist presented below is not intended to prescribe fixed indicators or universal key performance measures. Rather, it provides a structured set of reflective questions aligned with the seven ALTAI dimensions, reinterpreted through educational evaluation theory. The purpose is to guide institutional actors in verifying whether and how their policies on AI-based learning outcomes assessment address key governance areas. Each question highlights dimensions that require attention, while leaving to individual universities the responsibility of defining context-specific criteria, standards, and monitoring procedures.

Dimension (ALTAI)	Item Code	XAI-ED CAF Checklist Question
**1. Human agency & oversight**	1.1	Does the policy explicitly state that educators must retain final decision-making authority in AI-supported assessment processes?
	1.2	Does the policy establish formal procedures allowing students to appeal or contest AI-influenced assessment outcomes?
	1.3	Does the policy require meaningful human oversight to prevent fully automated grading without educator review?
	1.4	Does the policy mandate staff training on responsible oversight and limitations of AI-supported assessment systems?
**2. Technical robustness & safety**	2.1	Does the policy require evidence of validity, reliability, and accuracy for AI-supported assessment tools before deployment?
	2.2	Does the policy mandate that AI outputs demonstrate alignment with intended learning outcomes and assessment criteria?
	2.3	Does the policy establish procedures for testing, monitoring errors, and updating or decommissioning AI-supported assessment tools?

Dimension (ALTAI)	Item Code	XAI-ED CAF Checklist Question
	2.4	Does the policy require internal or external reviews and audits of AI-supported assessment systems?
**3. Privacy & data governance**	3.1	Does the policy explicitly reference compliance with GDPR or equivalent data protection principles for AI-supported assessment?
	3.2	Does the policy implement data minimization, retention limits, and lawful bases for processing in AI-supported assessment contexts?
	3.3	Does the policy guarantee students' rights to access, correction, and deletion of data used in AI-supported assessment?
	3.4	Does the policy assign clear responsibilities to a Data Protection Officer or equivalent role for AI-supported assessment data?
**4. Transparency**	4.1	Does the policy require mandatory disclosure when AI systems influence learning outcomes assessment or grading?
	4.2	Does the policy mandate provision of comprehensible explanations for AI-supported assessment decisions to all stakeholders?
	4.3	Does the policy ensure stakeholder access to information about assessment criteria, model limitations, and potential error sources?
	4.4	Does the policy establish communication protocols and support resources for non-technical users interpreting AI-supported assessment outcomes?
**5. Diversity, fairness & non-discrimination**	5.1	Does the policy explicitly prohibit discrimination and bias in AI-supported assessment systems?

Dimension (ALTAI)	Item Code	XAI-ED CAF Checklist Question
	5.2	Does the policy require systematic monitoring for bias and differential impacts across student groups, with mandatory corrective measures?
	5.3	Does the policy ensure accessibility and inclusion for disadvantaged or disabled students in AI-supported assessment processes?
	5.4	Does the policy mandate stakeholder consultation in the design, deployment, and evaluation of AI-supported assessment systems?
**6. Societal & environmental well-being**	6.1	Does the policy explicitly connect AI adoption in assessment to educational values such as inclusion, collaboration, trust, and learner autonomy?
	6.2	Does the policy address environmental implications of AI-supported assessment, including computational resource consumption and sustainability measures?
	6.3	Does the policy reference ethical codes or charters that promote human-centered education in relation to AI-supported assessment?
**7. Accountability**	7.1	Does the policy clearly define governance roles and responsibilities for AI-supported assessment implementation and oversight?
	7.2	Does the policy establish oversight committees or ethics boards responsible for monitoring AI-supported assessment and addressing grievances?
	7.3	Does the policy create mechanisms for stakeholder consultation, feedback collection, complaints processing, and redress procedures?

Dimension (ALTAI)	Item Code	XAI-ED CAF Checklist Question
	7.4	Does the policy mandate periodic review and updating of AI-supported assessment policies, including requirements for independent validation?

Scoring rubric (per item):

- **0 = No evidence found**
- **1 = Weak evidence** (reference present but vague, no supporting details)
- **2 = Partial implementation** (policy explicitly stated, limited operationalization)
- **3 = Documented implementation** (policy stated and supported by verifiable evidence)

Each dimension is scored as the mean of its items, then normalized on a 0-100 scale.

3 Detailed Coding Matrix with Consensus Scores and Qualitative Evidence.

University	Item	Score	Notes
UNITE	1.1	**1**	Teachers remain responsible, but no explicit clause guarantees final decision authority in AI-supported assessment.
UNITE	1.2	**0**	No appeal or contest procedures are defined for AI-influenced assessment outcomes.
UNITE	1.3	**2**	Meaningful human oversight is generally required, but fully automated grading is not explicitly prohibited.
UNITE	1.4	**2**	The university commits to staff training on GenAI; training is not compulsory nor specific to oversight in assessment.
UNITE	2.1	**0**	No requirements for validity, reliability, or accuracy testing of AI-supported assessment tools.
UNITE	2.2	**0**	No mandate to demonstrate alignment of AI outputs with intended learning outcomes or grading criteria.
UNITE	2.3	**0**	No lifecycle procedures (testing, error monitoring, decommissioning) are provided for AI tools in assessment.
UNITE	2.4	**0**	No internal or external reviews/audits are required for AI-supported assessment systems.

UNITE	3.1	**3**	Explicit GDPR compliance and DPO involvement are stated; assessment-specific details remain generic.
UNITE	3.2	**1**	Data minimization is encouraged (e.g., avoid personal data), but retention limits and lawful bases are not specified.
UNITE	3.3	**0**	Students' rights to access, rectification, or erasure in AI-assessment contexts are not articulated.
UNITE	3.4	**0**	The DPO role is not assigned explicit responsibilities for AI-supported assessment data.
UNITE	4.1	**1**	Disclosure focuses on students'/teachers' AI use in coursework; there is no institutional disclosure when AI influences grading.
UNITE	4.2	**0**	No requirement to provide comprehensible explanations for AI-supported assessment decisions.
UNITE	4.3	**1**	Some guidance on documenting prompts/outputs exists, but there is no structured access to criteria, model limits, or error sources.
UNITE	4.4	**1**	General training is offered, yet no dedicated communication protocols/support resources help non-technical users interpret AI-derived results.
UNITE	5.1	**1**	Fairness and bias risks are acknowledged, but there is no explicit non-discrimination clause for AI assessment systems.
UNITE	5.2	**0**	No framework for systematic bias monitoring or corrective measures across student groups.

UNITE	5.3	**1**	Accessibility is referenced in general terms; assessment-specific accommodations for disadvantaged/disabled students are not ensured.
UNITE	5.4	**0**	No stakeholder consultation mechanisms are mandated for the design or evaluation of AI-supported assessment.
UNITE	6.1	**2**	GenAI use is linked to educational values (e.g., trust, learner autonomy), but the connection to assessment contexts is indirect.
UNITE	6.2	**3**	Environmental impacts (energy/resource costs) are explicitly considered; references to assessment remain general.
UNITE	6.3	**3**	Ethical codes/charters (e.g., EU guidance) are referenced; ties to assessment are implicit rather than explicit.
UNITE	7.1	**0**	No governance roles or responsibilities are defined for AI-assessment implementation and oversight.
UNITE	7.2	**0**	No oversight committee or ethics board is designated to monitor AI-supported assessment.
UNITE	7.3	**0**	No complaint, feedback, or redress mechanisms are specified for AI-influenced assessment outcomes.
UNITE	7.4	**0**	No periodic review cycle or independent validation is mandated for AI-assessment policies/guidelines.

UNIBO	1.1	**2**	The policy states that educators maintain responsibility and discourages delegation of final grading to AI, though it is not a strong explicit mandate.
UNIBO	1.2	**0**	No procedures are defined for students to appeal or contest AI-influenced assessment outcomes.
UNIBO	1.3	**3**	Automated grading is explicitly precluded; human review is required.
UNIBO	1.4	**1**	The university promotes training on GenAI for staff, but it is generic and not mandatory for AI-assessment oversight.
UNIBO	2.1	**0**	No requirement for validity, reliability, or accuracy testing of AI-supported assessment tools.
UNIBO	2.2	**1**	Mentions relevance of AI-generated exercises to learning objectives, but no systematic alignment with assessment criteria.
UNIBO	2.3	**0**	No procedures for testing, error monitoring, or decommissioning of AI assessment systems.
UNIBO	2.4	**0**	No requirements for internal or external review/audit of AI-supported assessment.
UNIBO	3.1	**1**	Privacy cautions and references to rights are present, but GDPR compliance is not strongly articulated for assessment contexts.
UNIBO	3.2	**1**	Generic advice not to upload personal data reflects data minimization principles, but lacks rules on retention or lawful bases.

UNIBO	3.3	**0**	No mention of students' rights to access, correction, or erasure in AI-supported assessment contexts.
UNIBO	3.4	**1**	DPO is mentioned in general, but not with responsibilities specific to AI-supported assessment.
UNIBO	4.1	**0**	Disclosure is required for AI use by students/teachers, but not when AI influences grading decisions.
UNIBO	4.2	**0**	No guarantee of comprehensible explanations for AI-supported assessment outcomes.
UNIBO	4.3	**0**	Awareness of probabilistic model outputs is encouraged, but no access to assessment criteria, model limits, or error sources is provided.
UNIBO	4.4	**1**	Commitments to training and information exist, but no communication protocols to support interpretation of AI assessment outcomes.
UNIBO	5.1	**1**	Equity and inclusivity are highlighted; automation bias risks are noted, but no explicit non-discrimination clause exists.
UNIBO	5.2	**0**	No framework for systematic bias monitoring or corrective measures.
UNIBO	5.3	**1**	General references to inclusivity, but no accessibility guarantees in assessment workflows.
UNIBO	5.4	**0**	Community involvement is encouraged, but there is no stakeholder consultation specific to assessment systems.
UNIBO	6.1	**1**	Broad references to inclusion and trust, but no explicit link between values and AI assessment.

UNIBO	6.2	**1**	Environmental impacts of AI use are mentioned; application to assessment remains partial.
UNIBO	6.3	**1**	Cites ethical codes and frameworks, but references to assessment are indirect.
UNIBO	7.1	**1**	Institutional responsibilities are set, but no defined governance roles specific to AI assessment oversight.
UNIBO	7.2	**0**	No oversight committees or boards are designated for AI-supported assessment.
UNIBO	7.3	**1**	Feedback channels exist for AI in general, but no complaints or redress processes specific to assessment.
UNIBO	7.4	**0**	The policy pledges updates but does not establish periodic review or independent validation mechanisms.
UNIPR	1.1	**2**	Teachers retain responsibility for assessment decisions, though a strict clause on final decision authority is not fully explicit.
UNIPR	1.2	**0**	No appeal/contest procedures are defined for AI-influenced assessment outcomes.
UNIPR	1.3	**3**	Automated grading is effectively precluded and human review is required.
UNIPR	1.4	**0**	No mandate for staff training specific to responsible oversight of AI in assessment.

UNIPR	2.1	**0**	No requirements for validity, reliability, or accuracy testing of AI-supported assessment tools.
UNIPR	2.2	**0**	No obligation to demonstrate alignment of AI outputs with intended learning outcomes or grading criteria.
UNIPR	2.3	**0**	No lifecycle procedures (testing, error monitoring, decommissioning) for AI in assessment.
UNIPR	2.4	**0**	No internal/external review or audit requirements for AI-supported assessment systems.
UNIPR	3.1	**3**	Explicit GDPR compliance and DPO involvement are stated; assessment-specific details remain generic.
UNIPR	3.2	**1**	Data minimization is encouraged (e.g., avoid personal data), but rules on retention and lawful bases are not specified.
UNIPR	3.3	**0**	Students' rights to access, rectification, or erasure in AI-assessment contexts are not articulated.
UNIPR	3.4	**1**	The DPO is referenced; responsibilities for AI-assessment data are not clearly assigned.
UNIPR	4.1	**1**	Disclosure focuses on students'/teachers' AI use in coursework; no institutional disclosure when AI influences grading.
UNIPR	4.2	**0**	No requirement to provide comprehensible explanations for AI-supported assessment decisions.

UNIPR	4.3	**1**	Some guidance on documenting prompts/outputs exists; no structured access to criteria, model limits, or error sources.
UNIPR	4.4	**1**	General training is offered, but no dedicated communication protocols/support resources to interpret AI-derived results.
UNIPR	5.1	**1**	Fairness and bias risks are acknowledged, yet there is no explicit non-discrimination clause for AI assessment systems.
UNIPR	5.2	**0**	No framework for bias monitoring or corrective measures across student groups.
UNIPR	5.3	**0**	No measures ensure accessibility or inclusion of disadvantaged/disabled students in AI-supported assessment.
UNIPR	5.4	**0**	No stakeholder consultation mechanisms are mandated for AI-supported assessment.
UNIPR	6.1	**2**	GenAI use is linked to educational values (trust, learner autonomy), but the tie to assessment is indirect.
UNIPR	6.2	**0**	Environmental implications of AI are not addressed in assessment contexts.
UNIPR	6.3	**1**	Ethical codes/charters are referenced in broad terms; assessment-specific references are limited.
UNIPR	7.1	**0**	No governance roles or responsibilities are defined for AI-assessment implementation and oversight.

UNIPR	7.2	**0**	No oversight committee or ethics board is designated to monitor AI-supported assessment.
UNIPR	7.3	**0**	No complaint, feedback, or redress mechanisms are specified for AI-influenced assessment outcomes.
UNIPR	7.4	**2**	The guidelines commit to periodic updating, but do not require independent validation of AI-assessment policies.
UNIBG	1.1	**2**	Teachers retain responsibility for assessment decisions, though an explicit clause on final decision authority is not fully stated.
UNIBG	1.2	**0**	No appeal/contest procedures are defined for AI-influenced assessment outcomes.
UNIBG	1.3	**3**	Automated grading is effectively precluded and human review is required; one rater is slightly stricter due to wording.
UNIBG	1.4	**2**	Training on GenAI is committed institutionally, but it is not compulsory nor specific to oversight of AI in assessment.
UNIBG	2.1	**1**	Some concern for accuracy is acknowledged, yet there is no formal requirement for validity/reliability testing of assessment tools.
UNIBG	2.2	**1**	Partial recognition of alignment with learning outcomes exists, but no systematic mandate tied to grading criteria.

UNIBG	2.3	**1**	Lifecycle controls (testing/monitoring/decommissioning) are hinted at but not specified as procedures.
UNIBG	2.4	**0**	No internal/external review or audit requirements for AI-supported assessment systems.
UNIBG	3.1	**3**	Explicit GDPR compliance and DPO involvement are stated; assessment-specific details remain general.
UNIBG	3.2	**1**	Data minimization is encouraged (e.g., avoid personal data), but retention limits and lawful bases are not defined.
UNIBG	3.3	**0**	Students' rights to access, rectification, or erasure in AI-assessment contexts are not articulated.
UNIBG	3.4	**0**	No explicit assignment of DPO responsibilities for AI-assessment data.
UNIBG	4.1	**2**	Disclosure focuses on students'/teachers' AI use in coursework; no institutional disclosure when AI influences grading.
UNIBG	4.2	**0**	No requirement to provide comprehensible explanations of AI-supported assessment decisions.
UNIBG	4.3	**1**	Some guidance to record prompts/outputs exists, but no structured access to criteria, model limitations, or error sources.
UNIBG	4.4	**0**	No communication protocols or support resources to help non-technical users interpret AI-derived results.

UNIBG	5.1	**2**	Fairness and bias risks are highlighted; there is no explicit non-discrimination clause for AI assessment systems.
UNIBG	5.2	**0**	No framework for systematic bias monitoring or corrective actions across student groups.
UNIBG	5.3	**1**	Accessibility is referenced in general terms; assessment-specific accommodations are not ensured.
UNIBG	5.4	**0**	No stakeholder consultation mechanisms are mandated for AI-supported assessment.
UNIBG	6.1	**2**	GenAI use is linked to educational values (trust, autonomy); the connection to assessment is indirect.
UNIBG	6.2	**1**	Environmental implications are mentioned at a high level, without explicit link to assessment workflows.
UNIBG	6.3	**2**	Ethical codes/charters are referenced broadly; assessment-specific guidance is limited.
UNIBG	7.1	**1**	General institutional responsibilities are noted, but no governance roles specific to AI-assessment oversight.
UNIBG	7.2	**0**	No oversight committee or ethics board is designated to monitor AI-supported assessment.
UNIBG	7.3	**0**	No complaint, feedback, or redress mechanisms are specified for AI-influenced assessment outcomes.

UNIBG	7.4	**1**	The guidelines indicate updates over time, without a formal periodic review cycle or independent validation.
UNIMI	1.1	**3**	Educators' final decision authority in assessment is explicitly guaranteed; one rater is slightly stricter in interpretation.
UNIMI	1.2	**0**	No procedures are defined for students to appeal or contest AI-influenced assessment outcomes.
UNIMI	1.3	**3**	Fully automated grading is effectively precluded and meaningful human oversight is required; one rater credits it slightly lower due to wording.
UNIMI	1.4	**2**	Staff training on GenAI is planned/encouraged, but not compulsory nor specifically focused on oversight for assessment.
UNIMI	2.1	**1**	Basic attention to accuracy/reliability is noted, yet there is no formal requirement for validity/reliability testing of assessment tools.
UNIMI	2.2	**2**	Alignment of AI outputs to intended learning outcomes is addressed more clearly than average, though not as a full procedural mandate.
UNIMI	2.3	**1**	Lifecycle controls (testing/monitoring/decommissioning) are hinted at but not codified as procedures.
UNIMI	2.4	**1**	Some review intent is referenced, but no explicit internal/external audit requirement is set for AI-supported assessment.

UNIMI	3.1	**3**	Explicit GDPR compliance and DPO involvement are stated; assessment-specific provisions remain general.
UNIMI	3.2	**2**	Data minimization and related safeguards are addressed; retention limits and lawful bases are referenced but not fully operationalized.
UNIMI	3.3	**0**	Students' rights (access, rectification, erasure) in AI-assessment contexts are not articulated.
UNIMI	3.4	**1**	The DPO is referenced; responsibilities specific to AI-assessment data governance are not clearly assigned.
UNIMI	4.1	**3**	Strong disclosure rules: AI use impacting coursework/assessment must be declared; one rater gives slightly lower credit for institutional-vs-user disclosure nuance.
UNIMI	4.2	**1**	Some provisions support transparency, but there is no explicit right to a comprehensible explanation of AI-influenced assessment decisions.
UNIMI	4.3	**1**	Documentation of prompts/outputs and awareness of limitations are encouraged; structured access to criteria, model limits, and error sources is limited.
UNIMI	4.4	**1**	General training/info exists, but no dedicated communication protocols or support resources to help non-technical users interpret AI-derived results.
UNIMI	5.1	**2**	Fairness and non-discrimination are addressed more explicitly than average, though not fully operationalized for AI systems.

UNIMI	5.2	**1**	Bias monitoring is acknowledged at a high level; there is no systematic requirement with corrective measures.
UNIMI	5.3	**2**	Accessibility/inclusion considerations are present, with partial safeguards for assessment; implementation details are limited.
UNIMI	5.4	**1**	Stakeholder consultation is encouraged but not mandated nor structured for AI-supported assessment.
UNIMI	6.1	**2**	GenAI adoption is linked to educational values (e.g., inclusion, learner autonomy); the tie to assessment contexts is indirect.
UNIMI	6.2	**2**	Environmental implications (energy/resources) are recognized, without assessment-specific operational measures.
UNIMI	6.3	**3**	Ethical codes and international charters (e.g., EU) are explicitly referenced; links to assessment practice are implicit.
UNIMI	7.1	**2**	Governance responsibilities are described at a higher level; assessment-specific oversight roles are not fully defined.
UNIMI	7.2	**2**	Some oversight arrangements exist, though formal committees/boards dedicated to AI assessment are not clearly instituted.
UNIMI	7.3	**1**	General feedback/complaint channels exist; no redress mechanism specific to AI-influenced assessment outcomes.

UNIMI	7.4	**1**	There is a commitment to update guidance over time, but no formal periodic review cycle or independent validation requirement.
POLIMI	1.1	**1**	Teachers remain responsible, but there is no explicit clause guaranteeing educators' final decision authority in AI-supported assessment.
POLIMI	1.2	**0**	No procedures are defined for students to appeal or contest AI-influenced assessment outcomes.
POLIMI	1.3	**1**	Human oversight is implied, yet fully automated grading is not expressly prohibited.
POLIMI	1.4	**1**	Staff training on GenAI is encouraged but not compulsory nor specifically focused on oversight for assessment.
POLIMI	2.1	**0**	No requirements for validity, reliability, or accuracy testing of AI-supported assessment tools.
POLIMI	2.2	**0**	No obligation to demonstrate alignment of AI outputs with intended learning outcomes or grading criteria.
POLIMI	2.3	**1**	Lifecycle controls (testing/monitoring/decommissioning) are hinted at but not defined as procedures.
POLIMI	2.4	**0**	No internal or external review/audit requirements for AI-supported assessment systems.
POLIMI	3.1	**2**	GDPR compliance and DPO involvement are stated; provisions for assessment are generic.

POLIMI	3.2	**1**	Data minimization is encouraged (e.g., avoid personal data), but retention limits and lawful bases are not specified.
POLIMI	3.3	**0**	Students' rights to access, rectification, or erasure in AI-assessment contexts are not articulated.
POLIMI	3.4	**0**	No explicit assignment of DPO responsibilities for AI-assessment data governance.
POLIMI	4.1	**1**	Disclosure focuses on students'/teachers' AI use in coursework; there is no institutional disclosure when AI influences grading.
POLIMI	4.2	**0**	No requirement to provide comprehensible explanations for AI-supported assessment decisions.
POLIMI	4.3	**0**	No guarantees of stakeholder access to assessment criteria, model limitations, or error sources.
POLIMI	4.4	**0**	No communication protocols or support resources for non-technical users interpreting AI-derived results.
POLIMI	5.1	**0**	No explicit non-discrimination clause addressing AI-supported assessment systems.
POLIMI	5.2	**0**	No framework for bias monitoring or corrective measures across student groups.
POLIMI	5.3	**0**	No provisions to ensure accessibility/inclusion for disadvantaged or disabled students in AI-supported assessment.

POLIMI	5.4	**0**	No stakeholder consultation mechanisms are mandated for AI-supported assessment.
POLIMI	6.1	**1**	GenAI use is linked to educational values (e.g., trust, autonomy), but the connection to assessment contexts is indirect.
POLIMI	6.2	**0**	Environmental implications of AI are not addressed in assessment workflows.
POLIMI	6.3	**1**	Ethical codes/charters are referenced broadly; assessment-specific guidance is limited.
POLIMI	7.1	**0**	No governance roles or responsibilities are defined for AI-assessment implementation and oversight.
POLIMI	7.2	**0**	No oversight committee or ethics board is designated to monitor AI-supported assessment.
POLIMI	7.3	**0**	No complaint, feedback, or redress mechanisms are specified for AI-influenced assessment outcomes.
POLIMI	7.4	**1**	There is a commitment to update guidance over time, but no formal periodic review cycle or independent validation.
UNICAM	1.1	**2**	Teachers retain responsibility for assessment; however, an explicit clause guaranteeing final decision authority is not fully stated.
UNICAM	1.2	**0**	No procedures are defined for students to appeal or contest AI-influenced assessment outcomes.

UNICAM	1.3	**3**	Fully automated grading is effectively precluded and meaningful human oversight is required; one rater is slightly stricter due to wording.
UNICAM	1.4	**1**	Staff training on GenAI is encouraged, but it is not compulsory nor specifically focused on oversight for assessment.
UNICAM	2.1	**1**	Some attention to accuracy/reliability appears, yet there is no formal requirement for validity/reliability testing of assessment tools.
UNICAM	2.2	**0**	No mandate exists to demonstrate alignment of AI outputs with intended learning outcomes or grading criteria.
UNICAM	2.3	**1**	Lifecycle controls (testing/monitoring/decommissioning) are hinted at but not defined as procedures.
UNICAM	2.4	**2**	Some internal review intent is referenced; however, there is no explicit institutional audit requirement for AI-supported assessment.
UNICAM	3.1	**3**	Explicit GDPR compliance and DPO involvement are stated; assessment-specific provisions remain general.
UNICAM	3.2	**1**	Data minimization is encouraged (e.g., avoid personal data), but retention limits and lawful bases are not specified.
UNICAM	3.3	**0**	Students' rights (access, rectification, erasure) in AI-assessment contexts are not articulated.

UNICAM	3.4	**0**	No explicit assignment of DPO responsibilities for AI-assessment data governance.
UNICAM	4.1	**1**	Disclosure focuses on students'/teachers' AI use in coursework; there is no institutional disclosure when AI influences grading.
UNICAM	4.2	**0**	No requirement to provide comprehensible explanations for AI-supported assessment decisions.
UNICAM	4.3	**0**	No guarantees of stakeholder access to assessment criteria, model limitations, or error sources.
UNICAM	4.4	**0**	No communication protocols or support resources are defined for non-technical users interpreting AI-derived results.
UNICAM	5.1	**0**	No explicit non-discrimination clause addressing AI-supported assessment systems.
UNICAM	5.2	**0**	No framework for bias monitoring or corrective measures across student groups.
UNICAM	5.3	**0**	No provisions to ensure accessibility/inclusion for disadvantaged or disabled students in AI-supported assessment.
UNICAM	5.4	**0**	No stakeholder consultation mechanisms are mandated for AI-supported assessment.
UNICAM	6.1	**1**	GenAI use is linked to educational values (e.g., trust, autonomy); the connection to assessment contexts is indirect.
UNICAM	6.2	**0**	Environmental implications of AI are not addressed in assessment workflows.

UNICAM	6.3	**2**	Ethical codes/charters are referenced explicitly; links to assessment practice are mostly implicit.
UNICAM	7.1	**1**	General institutional responsibilities are noted, but no governance roles specific to AI-assessment oversight are defined.
UNICAM	7.2	**1**	Some oversight intent is mentioned, yet no dedicated ethics board/committee for AI-supported assessment is clearly instituted.
UNICAM	7.3	**0**	No complaint, feedback, or redress mechanisms are specified for AI-influenced assessment outcomes.
UNICAM	7.4	**2**	There is a commitment to update guidance over time, but no formal periodic review cycle or independent validation is set.
UNIPO	1.1	**3**	Educators’ final decision authority is explicitly guaranteed; one rater is slightly stricter in interpretation.
UNIPO	1.2	**1**	Some recognition of students’ ability to contest outcomes exists, but no formalized appeal procedure is set.
UNIPO	1.3	**3**	Fully automated grading is precluded, with meaningful human oversight explicitly required.
UNIPO	1.4	**2**	Faculty training on responsible GenAI use is committed; not compulsory nor entirely specific to oversight in assessment.

UNIPO	2.1	**2**	Requirements for validity/reliability are addressed more explicitly than average, though not formalized as a process.
UNIPO	2.2	**1**	Partial attention to alignment with learning outcomes is present; not mandated systematically.
UNIPO	2.3	**2**	Monitoring and updating of AI tools are acknowledged; procedural details remain limited.
UNIPO	2.4	**2**	Internal/external review is referenced but not mandated as a structured audit requirement.
UNIPO	3.1	**3**	Explicit GDPR compliance and DPO involvement are stated; assessment-related provisions are general.
UNIPO	3.2	**3**	Data minimization, lawful bases, and retention limits are explicitly referenced, though implementation may vary.
UNIPO	3.3	**2**	Students' rights (access, rectification, erasure) are mentioned, but not systematically ensured in AI-assessment contexts.
UNIPO	3.4	**1**	DPO is referenced; explicit responsibilities for AI-assessment data governance are not clearly defined.
UNIPO	4.1	**1**	Disclosure obligations exist, but are not directly tied to AI's role in grading decisions.
UNIPO	4.2	**1**	Some transparency provisions exist, but no explicit right to comprehensible explanations of AI-influenced outcomes.

UNIPO	4.3	**1**	Stakeholders are encouraged to record prompts/outputs; access to criteria, model limits, or error sources is not structured.
UNIPO	4.4	**1**	General training/support exists, but no dedicated communication protocols for non-technical users.
UNIPO	5.1	**2**	Fairness and non-discrimination are addressed, though not fully operationalized for AI assessment.
UNIPO	5.2	**2**	Bias monitoring and impacts across groups are acknowledged, though corrective mechanisms are not mandatory.
UNIPO	5.3	**1**	Accessibility and inclusion are noted, but assessment-specific accommodations are not explicitly defined.
UNIPO	5.4	**1**	Stakeholder consultation is encouraged but not structured nor mandated for AI assessment.
UNIPO	6.1	**2**	GenAI adoption is linked to educational values (trust, autonomy, inclusion); the tie to assessment remains indirect.
UNIPO	6.2	**0**	Environmental implications of AI are not addressed in assessment contexts.
UNIPO	6.3	**2**	Ethical codes/charters are explicitly referenced; links to assessment practice are mostly implicit.
UNIPO	7.1	**3**	Governance roles are clearly defined; not all provisions are assessment-specific.

UNIPO	7.2	**2**	Oversight committees/boards are acknowledged; their role in AI-supported assessment is not fully detailed.
UNIPO	7.3	**1**	General complaint/feedback channels exist; no redress mechanisms are specific to AI-influenced assessment.
UNIPO	7.4	**2**	Commitment to review and update policies is present, though periodic cycles and independent validation are not mandated.
UNIFI	1.1	**3**	Educators' final decision authority in assessment is explicitly guaranteed; one rater scores slightly lower due to wording nuance.
UNIFI	1.2	**0**	No procedures are defined for students to appeal or contest AI-influenced assessment outcomes.
UNIFI	1.3	**3**	Fully automated grading is precluded and meaningful human oversight is required; one rater assigns slightly less credit.
UNIFI	1.4	**1**	Staff training on GenAI is encouraged, but it is not compulsory nor specifically focused on oversight for assessment.
UNIFI	2.1	**1**	Some attention to accuracy/reliability appears, yet there is no formal requirement for validity/reliability testing of assessment tools.
UNIFI	2.2	**1**	Partial recognition that outputs should match learning outcomes; no systematic mandate tied to grading criteria.

UNIFI	2.3	**0**	No lifecycle procedures (testing, error monitoring, decommissioning) for AI in assessment.
UNIFI	2.4	**0**	No internal or external review/audit requirements for AI-supported assessment systems.
UNIFI	3.1	**2**	GDPR compliance and DPO support are stated; assessment-specific provisions remain general.
UNIFI	3.2	**2**	Data minimization and related safeguards are addressed; retention limits/lawful bases are referenced but not fully operationalized.
UNIFI	3.3	**0**	Students' rights (access, rectification, erasure) in AI-assessment contexts are not articulated.
UNIFI	3.4	**0**	No explicit assignment of DPO responsibilities for AI-assessment data governance.
UNIFI	4.1	**2**	Disclosure duties exist for AI use in coursework; no institutional disclosure when AI influences grading decisions.
UNIFI	4.2	**1**	Some transparency provisions exist, but no explicit right to a comprehensible explanation of AI-influenced outcomes.
UNIFI	4.3	**2**	Documentation of prompts/outputs and awareness of limitations are encouraged; structured access to criteria/model limits remains limited.
UNIFI	4.4	**1**	General training/info exists, but no dedicated communication protocols or support resources for non-technical users.

UNIFI	5.1	**2**	Fairness and non-discrimination are addressed more explicitly than average, though not fully operationalized for AI systems.
UNIFI	5.2	**0**	No framework for systematic bias monitoring or corrective measures across student groups.
UNIFI	5.3	**3**	Accessibility/inclusion are strongly emphasized with partial safeguards for assessment; implementation details are limited.
UNIFI	5.4	**0**	No stakeholder consultation mechanisms are mandated for AI-supported assessment.
UNIFI	6.1	**2**	GenAI adoption is linked to educational values (inclusion, learner autonomy); the tie to assessment is indirect.
UNIFI	6.2	**2**	Environmental implications (energy/resources) are recognized, without assessment-specific operational measures.
UNIFI	6.3	**2**	Ethical codes/charters are referenced; links to assessment practice are largely implicit.
UNIFI	7.1	**1**	General institutional responsibilities are noted, but no governance roles specific to AI-assessment oversight are defined.
UNIFI	7.2	**0**	No oversight committee or ethics board is designated to monitor AI-supported assessment.
UNIFI	7.3	**0**	No complaint, feedback, or redress mechanisms are specified for AI-influenced assessment outcomes.

UNIFI	7.4	**2**	Commitment to review/update policies is present, but no formal periodic cycle or independent validation is mandated.
UNISI	1.1	**2**	Educators are confirmed as responsible in assessment, though the policy does not always phrase it as exclusive final authority.
UNISI	1.2	**0**	No appeal or contestation mechanism is foreseen for AI-influenced assessment outcomes.
UNISI	1.3	**2**	Meaningful human oversight is required and automation is discouraged; wording remains somewhat generic.
UNISI	1.4	**2**	Training for staff on GenAI use is promoted, but not fully specified as mandatory oversight preparation.
UNISI	2.1	**1**	Some references to accuracy and reliability are present, but without formal requirements for validation of assessment tools.
UNISI	2.2	**0**	No clear mandate that AI outputs must align with learning outcomes and grading criteria.
UNISI	2.3	**1**	Monitoring and corrective actions are mentioned in principle, but no structured procedures for testing/updating tools exist.
UNISI	2.4	**0**	No internal or external audits are required for AI systems in assessment.

UNISI	3.1	**2**	GDPR compliance is explicitly required; coverage is broad but not tailored to AI assessment contexts.
UNISI	3.2	**1**	Data minimization and lawful bases are encouraged indirectly; retention rules are not fully detailed.
UNISI	3.3	**0**	Students' rights to access, correction, or deletion of assessment data are not addressed.
UNISI	3.4	**0**	No clear allocation of responsibilities to the DPO for AI assessment data.
UNISI	4.1	**2**	Disclosure obligations exist for AI use, but institutional communication on grading influence is not mandated.
UNISI	4.2	**1**	Some guidance toward transparency is offered, though no explicit right to comprehensible explanations is guaranteed.
UNISI	4.3	**1**	Stakeholders are advised to reflect on limitations; no structured information access on models or errors is provided.
UNISI	4.4	**1**	Training and general awareness initiatives are included, without clear support resources for non-technical users.
UNISI	5.1	**1**	Fairness and equity principles are acknowledged, but without an explicit ban on discrimination in AI-supported assessment.
UNISI	5.2	**0**	No monitoring process for bias or corrective measures across groups is established.

UNISI	5.3	**0**	Accessibility and inclusion for disadvantaged students are not addressed in the assessment context.
UNISI	5.4	**1**	Consultation and collaboration are encouraged broadly, but no explicit stakeholder involvement is required in assessment design.
UNISI	6.1	**1**	AI adoption is linked to educational values like autonomy and responsibility, but connections to assessment remain indirect.
UNISI	6.2	**0**	Environmental sustainability issues are not addressed in assessment-related AI use.
UNISI	6.3	**1**	References to ethical codes are present, though not tied specifically to AI in assessment.
UNISI	7.1	**1**	General governance responsibilities are indicated, but no specific roles for AI-assessment oversight are defined.
UNISI	7.2	**0**	No committee or ethics board is tasked with monitoring AI assessment practices.
UNISI	7.3	**1**	Feedback and complaint mechanisms exist in general academic regulations, but no AI-specific measures are foreseen.
UNISI	7.4	**1**	Commitment to update guidelines is explicit; periodic cycles or independent validation are not specified.
UNITN	1.1	**2**	Educators are responsible for assessment decisions, though an explicit clause on exclusive final authority is not fully stated.

UNITN	1.2	**0**	No appeal/contest procedures are provided for AI-influenced assessment outcomes.
UNITN	1.3	**2**	Meaningful human oversight is required and automation discouraged; the prohibition is not fully explicit.
UNITN	1.4	**1**	Staff training on GenAI is encouraged, but it is neither compulsory nor specifically focused on assessment oversight.
UNITN	2.1	**1**	Some attention to accuracy/reliability appears, without formal validity/reliability requirements for assessment tools.
UNITN	2.2	**0**	No mandate that AI outputs align with learning outcomes and grading criteria.
UNITN	2.3	**1**	Lifecycle controls (testing/monitoring/decommissioning) are hinted at but not defined as procedures.
UNITN	2.4	**0**	No internal or external reviews/audits are required for AI-supported assessment systems.
UNITN	3.1	**2**	GDPR compliance and DPO support are stated; assessment-specific provisions remain general.
UNITN	3.2	**1**	Data minimization is encouraged (e.g., avoid personal data), but retention and lawful-basis rules are not specified.
UNITN	3.3	**0**	Students' rights (access, rectification, erasure) in AI-assessment contexts are not articulated.
UNITN	3.4	**0**	No explicit assignment of DPO responsibilities for AI-assessment data governance.

UNITN	4.1	**2**	Disclosure duties exist for AI use in coursework; institutional disclosure when AI influences grading is not mandated.
UNITN	4.2	**1**	Some transparency provisions exist, but no explicit right to comprehensible explanations of AI-influenced outcomes.
UNITN	4.3	**0**	No guarantees of stakeholder access to assessment criteria, model limitations, or error sources.
UNITN	4.4	**0**	No communication protocols or support resources for non-technical users interpreting AI-derived results.
UNITN	5.1	**1**	Fairness and bias risks are acknowledged; there is no explicit non-discrimination clause for AI assessment systems.
UNITN	5.2	**0**	No framework for systematic bias monitoring or corrective measures across student groups.
UNITN	5.3	**0**	No provisions ensure accessibility or inclusion for disadvantaged/disabled students in AI-supported assessment.
UNITN	5.4	**0**	No stakeholder consultation mechanisms are mandated for AI-supported assessment.
UNITN	6.1	**1**	GenAI use is linked to educational values (e.g., autonomy, trust); the connection to assessment contexts is indirect.

UNITN	6.2	**1**	Environmental implications are mentioned at a high level, without assessment-specific operational measures.
UNITN	6.3	**1**	Ethical codes/charters are referenced broadly; links to assessment practice are mostly implicit.
UNITN	7.1	**1**	General governance responsibilities are indicated, but no roles specific to AI-assessment oversight are defined.
UNITN	7.2	**0**	No committee or ethics board is tasked with monitoring AI-supported assessment.
UNITN	7.3	**0**	No complaint, feedback, or redress mechanisms are specified for AI-influenced assessment outcomes.
UNITN	7.4	**1**	Commitment to update guidance over time is stated, but no formal periodic review cycle or independent validation is mandated.
UNISTRAPG	1.1	**2**	The policy emphasizes that teachers maintain responsibility and discourages AI substitution in grading.
UNISTRAPG	1.2	**0**	No procedures exist for students to appeal or contest AI-influenced assessment results.
UNISTRAPG	1.3	**2**	Automated grading is explicitly precluded; meaningful human oversight is consistently required.
UNISTRAPG	1.4	**1**	The guidelines commit to training initiatives for staff, though they are not yet structured or mandatory.

UNISTRAPG	2.1	**3**	The policy requires validity checks for certain AI use cases, especially code-related tasks.
UNISTRAPG	2.2	**0**	No provisions ensure systematic alignment of AI outputs with learning outcomes or assessment criteria.
UNISTRAPG	2.3	**3**	Procedures for testing, error monitoring, and decommissioning are clearly established.
UNISTRAPG	2.4	**2**	Internal audit and review measures are required for AI tools in teaching and assessment.
UNISTRAPG	3.1	**3**	The guidelines explicitly require GDPR compliance and involve the DPO.
UNISTRAPG	3.2	**2**	References to minimization and protection principles are present, though concrete limits on retention or lawful bases are not detailed.
UNISTRAPG	3.3	**1**	Mentions of data subject rights are generic.
UNISTRAPG	3.4	**1**	The DPO is mentioned but roles are not detailed for assessment.
UNISTRAPG	4.1	**2**	The policy requires students to declare AI use in assignments.
UNISTRAPG	4.2	**3**	The guidelines clearly encourage transparency in outputs.
UNISTRAPG	4.3	**2**	Stakeholders are encouraged to record prompts/outputs, which provides partial access to information on limitations and risks.

UNISTRAPG	4.4	**2**	Workshops and training exist to help students and staff interpret AI outcomes, but no formal communication protocols are defined.
UNISTRAPG	5.1	**3**	The document explicitly refers to fairness and warns against bias.
UNISTRAPG	5.2	**3**	Monitoring for bias and inequity is encouraged, corrective measures are codified.
UNISTRAPG	5.3	**0**	Some mention of accessibility values is included, but no provisions for disadvantaged or disabled students in AI assessment.
UNISTRAPG	5.4	**1**	Interdisciplinary collaboration and pilots are encouraged, but no mandatory stakeholder consultation processes are defined.
UNISTRAPG	6.1	**2**	The guidelines connect GenAI adoption to values of trust and collaboration; however, ties to assessment contexts are indirect.
UNISTRAPG	6.2	**2**	Environmental impacts of AI are recognized, but not specifically framed within assessment.
UNISTRAPG	6.3	**2**	The policy references ethical codes and international guidelines (EU AI Act, HLEG), though explicit ties to assessment are limited.
UNISTRAPG	7.1	**2**	High-level governance responsibilities are set, though no detailed oversight roles for assessment are defined.
UNISTRAPG	7.2	**1**	Mentions oversight in general, but no specific ethics boards or committees for AI-supported assessment.

UNISTRAPG	7.3	**1**	General feedback mechanisms exist via academic integrity procedures, but no redress mechanisms specific to AI in assessment.
UNISTRAPG	7.4	**2**	The guidelines commit to regular updates as technologies evolve, though no independent validation is required.
UNIVE	1.1	**0**	The policy stresses that teachers remain responsible, but it does not explicitly guarantee final decision authority in AI-supported assessment.
UNIVE	1.2	**0**	No procedures exist for students to appeal or contest AI-influenced assessment outcomes.
UNIVE	1.3	**0**	The text discourages reliance on AI detectors and emphasizes teacher verification, but it does not explicitly prohibit fully automated grading.
UNIVE	1.4	**1**	The university commits to staff training on GenAI, but training is general and not specific to oversight of AI-supported assessment.
UNIVE	2.1	**0**	There is limited reference to accuracy concerns (e.g., coding bug detection), but no structured requirement for validity or reliability of assessment tools.
UNIVE	2.2	**0**	No mandate exists to ensure AI outputs align with intended learning outcomes or assessment criteria.
UNIVE	2.3	**0**	No procedures are defined for testing, error monitoring, or decommissioning of AI-supported assessment tools.

UNIVE	2.4	**0**	No requirements for internal or external review or audit of AI-supported assessment systems.
UNIVE	3.1	**2**	GDPR compliance is explicitly referenced and the DPO is mentioned, although responsibilities specific to AI in assessment are not described.
UNIVE	3.2	**1**	The policy cautions against uploading personal data, reflecting minimization principles, but does not specify retention or lawful basis rules.
UNIVE	3.3	**0**	Students' rights to access, correction, or deletion of data are not articulated.
UNIVE	3.4	**1**	The DPO is referenced, but responsibilities regarding AI-supported assessment are not clearly assigned.
UNIVE	4.1	**1**	Students must disclose use of GenAI in coursework; however, there is no institutional disclosure when AI influences grading.
UNIVE	4.2	**1**	Disclosure provisions exist, but no structured requirement for providing comprehensible explanations of AI-supported assessment outcomes.
UNIVE	4.3	**0**	No guarantees are made for stakeholder access to criteria, model limitations, or error sources.
UNIVE	4.4	**0**	No communication protocols or support resources are defined for non-technical users interpreting AI-driven assessment outcomes.
UNIVE	5.1	**1**	The guidelines highlight risks of automation bias and reference equity, but there is no explicit non-discrimination clause for AI assessment.

UNIVE	5.2	**0**	No provisions require monitoring for bias or corrective measures across student groups.
UNIVE	5.3	**0**	No measures ensure accessibility or inclusion of disadvantaged or disabled students in AI-supported assessment.
UNIVE	5.4	**0**	No stakeholder consultation mechanisms are established for AI-supported assessment.
UNIVE	6.1	**1**	The policy links GenAI use to values such as originality and equity; the connection to assessment contexts is indirect.
UNIVE	6.2	**1**	Environmental impacts are mentioned in general terms; no explicit tie to AI-supported assessment.
UNIVE	6.3	**1**	References are made to ethical codes and EU guidelines, but they are broad and not assessment-specific.
UNIVE	7.1	**0**	No governance roles or responsibilities are defined for AI-supported assessment oversight.
UNIVE	7.2	**0**	No oversight committees or ethics boards are designated to monitor AI-supported assessment.
UNIVE	7.3	**0**	No mechanisms are provided for feedback, complaints, or redress regarding AI in assessment.
UNIVE	7.4	**0**	No commitments exist for periodic review or independent validation of AI-supported assessment policies.

UNINA	1.1	**0**	No official UNINA policy on GenAI in assessment has been located; no provisions regarding final decision-making authority.
UNINA	1.2	**0**	No policy available; no evidence of appeal or contest procedures related to AI-influenced assessment outcomes.
UNINA	1.3	**0**	No institutional requirement ensuring human oversight over automated grading has been identified.
UNINA	1.4	**0**	No commitments to staff training specific to AI-supported assessment oversight found.
UNINA	2.1	**0**	No pre-deployment requirements regarding validity, reliability, or accuracy of AI tools are defined.
UNINA	2.2	**0**	No evidence that AI outputs must align with learning outcomes or assessment criteria.
UNINA	2.3	**0**	No institutional procedures regarding testing, error monitoring, or decommissioning of AI systems.
UNINA	2.4	**0**	No audit or review mechanisms specified in available UNINA sources.
UNINA	3.1	**0**	No explicit mention of GDPR compliance in AI-supported assessment contexts found.
UNINA	3.2	**0**	No references to minimization, retention limits, or lawful bases for AI-supported assessment data.
UNINA	3.3	**0**	No articulation of data subject rights related to AI use in assessments identified.

UNINA	3.4	**0**	No clear assignment of Data Protection Officer roles in relation to AI-supported assessment.
UNINA	4.1	**0**	No disclosure rules requiring notification when AI contributes to assessment outcomes.
UNINA	4.2	**0**	No provisions requiring explanations of AI-influenced grading decisions.
UNINA	4.3	**0**	No obligations to provide information about AI model limitations or error sources.
UNINA	4.4	**0**	No communication protocols or support resources identified.
UNINA	5.1	**0**	No explicit clauses prohibiting discrimination or bias in AI-supported assessments.
UNINA	5.2	**0**	No monitoring or corrective measures for bias in AI systems identified.
UNINA	5.3	**0**	No policy references ensuring accessibility or inclusion in AI-supported assessments.
UNINA	5.4	**0**	No requirement for stakeholder consultation regarding AI use in assessment.
UNINA	6.1	**0**	No available documentation linking AI adoption in assessment to educational values.
UNINA	6.2	**0**	No references to environmental or sustainability considerations in AI assessment.
UNINA	6.3	**0**	No references to UNESCO, EU, or other ethical codes in relation to AI in assessment.

UNINA	7.1	**0**	No governance roles defined for AI-supported assessment.
UNINA	7.2	**0**	No oversight bodies designated for AI assessment monitoring.
UNINA	7.3	**0**	No mechanisms provided for feedback, complaints, or redress regarding AI in assessment.
UNINA	7.4	**0**	No commitment to review or independent validation of AI policies in assessment.
